# Mechanotransductive Osteogenesis Through Microarchitectural Stabilization in an Injectable Hydrogel–Mineral System

**DOI:** 10.3390/jfb17080389

**Published:** 2026-08-06

**Authors:** Young K. Kim, Wanting Niu, Christopher J. Love, Myron Spector

**Affiliations:** 1 Department of Prosthodontics, Harvard School of Dental Medicine, Boston, MA 02115, USA; 2 Tissue Engineering/Regenerative Medicine, VA Boston Healthcare System, Boston, MA 02130, USA; wanting.niu@regiscollege.edu (W.N.); cjlove.mit@gmail.com (C.J.L.); mspector@bwh.harvard.edu (M.S.); 3 Department of Prosthodontics, NYU College of Dentistry, New York, NY 10010, USA; 4 Department of Mechanical Engineering, Massachusetts Institute of Technology, Cambridge, MA 02139, USA; 5 Orthopedic Surgery, Brigham and Women’s Hospital, Harvard Medical School, Boston, MA 02115, USA

**Keywords:** mechanotransduction, injectable hydrogel, bone regeneration, calcium phosphate, mesenchymal stem cells, osteogenesis, microarchitecture, hydrogel–mineral composite, mechanical stabilization, tissue engineering

## Abstract

In the contemporary era of minimally invasive surgery, injectable biomaterial scaffolds have demonstrated significant potential in bone tissue engineering (BTE). Completely injectable hydrogel substratum with microscale bone graft particulates delivered through a needle-shaped orifice shifts a new paradigm for surgical interventions in clinical settings. Despite growing interest in biopolymer-based BTE systems, clinically applicable delivery platforms and a mechanistic understanding of cell–material interactions remain limited. This study developed a dual-syringe auto-mix system capable of generating an in situ cross-linking hydrogel–mineral construct composed of gelatin–hydroxyphenyl propionic acid, hyaluronic acid–tyramine, horseradish peroxidase, hydrogen peroxide, and calcium phosphate particles of varying sizes. Material distribution, rheological and mechanical properties, and swelling were characterized. Goat bone marrow-derived mesenchymal stem cells served as the basis for examining how the composite affected cell viability, morphology, proliferation, contractility, osteogenic differentiation, mineralization, and chemotactic behavior. To determine whether these biological findings were supported mechanically, an ex vivo cone-beam computed tomography model was used to evaluate volumetric stability and resistance to deformation at the graft–host interface. Cross-linking established a stable internal microarchitecture while remaining compatible with cell viability and nutrient-dependent survival. Formation of the gelatin–hyaluronan (GH) network significantly increased the storage modulus relative to gelatin (G) alone, whereas subsequent calcium phosphate incorporation (GH-CP) preserved this mechanical competence while attenuating the volumetric expansion of GH. These physical characteristics were accompanied by more organized cell morphology, enhanced osteogenic differentiation, and mineral deposition throughout a larger portion of the matrix. *Heterogeneous interpenetrating gap striation* (HIGS) appeared in regions of cellular aggregation and matrix deposition, and a new conceptualization of the osteogenic phenomenon, termed *cling osteogenesis*, has been proposed. These outcomes support an intricate relationship between early mechanical stabilization, mechanotransduction, and osteogenesis in injectable hydrogel–mineral systems.

## 1. Introduction

### 1.1. Mechanical Instability as a Fundamental Barrier to Bone Regeneration

Bone regeneration is a mechanically governed process in which the physical properties of the surrounding microenvironment critically shape cellular fate dynamics [1,2,3]. During the earliest phases of healing, regenerating tissues are exposed to dynamic compressive and tensile forces arising from wound-healing expansion, periosteal tension, soft-tissue recoil, and surgical wound closure. These forces regulate cytoskeletal organization, focal adhesion maturation, and downstream mechanotransductive signaling pathways, consequently influencing osteogenic lineage commitment [4,5,6,7,8,9,10]. When mechanically vulnerable conditions predominate during this early window, regenerative trajectories are compromised before osteogenesis is effectively initiated.

An emerging body of work has recognized that mechanical cues in bone regeneration are not constant but can be modulated by controlled, time-dependent loading [11,12]. Intermittent tensocompressive strategies—such as those achieved through retightenable periosteal or sling-based suturing—illustrate how gradual modulation of periosteal tensocompression can influence local mechanosensitivity during healing in clinical settings [13]. These empirical observations emphasize the potential importance of temporal tuning of mechanical signals in osteogenesis, beyond a single fixed mechanical state.

The ability of a graft to remain mechanically stable during early healing remains a fundamental requirement for successful bone regeneration. Structural rigidity at this stage provides the conditions needed for osteogenic differentiation and matrix mineralization to proceed [14,15,16]. The problem is amplified in non-contained and partially contained defects due to its architecturally insufficient anatomical morphology to stabilize the graft against such a destructive equilibrium of physiologic inertia. As a result, calcium phosphate grafts with favorable osteoconductive characteristics may still undergo displacement or deformation, largely because the particles themselves possess limited intrinsic cohesion and are continually influenced by the surrounding periosteal apparatus [17,18,19,20].

Early mechanical failure—graft micromotion, structural collapse, or loss of space maintenance—remains one of the major yet often overlooked reasons for unsuccessful bone regeneration. These challenges highlight the need for regenerative approaches that provide sufficient mechanical stability while still allowing cells to respond appropriately to mechanical cues during the earliest stages of healing.

### 1.2. Injectable Bone Grafting: Syringe-Assisted Extrusion and Translational Limitations

Injectable bone augmentation has been widely adopted as an alternative to block grafting and manual packing of particulate fillers. In such emerging surgical practice, many of these interventions rely on syringe-assisted extrusion of particulate bone grafts, often mixed with saline or blood, to facilitate placement into irregular or confined defects. This approach offers improved handling and conformability compared with standard techniques for layering particulates, but it remains essentially dependent on mechanical compaction and extensive surgical access [16,21].

When particulate grafts are used through a syringe, adequate flap reflection is still required to position the delivery tip and distribute the graft throughout the defect [22]. Delivery of particulate material via a relatively large-bore syringe frequently determined the extent of surgical exposure, regardless of defect morphology. Under these conditions, the operative field remained similar to that required for conventional grafting, particularly in anatomically or esthetically demanding regions. A further limitation is that even among currently available truly injectable hydrogel systems, there remain no clinically established modules that efficiently incorporate particulate bone graft materials into a fully injectable construct capable of supporting a genuinely minimally invasive augmentation workflow [23,24,25].

These mechanical limitations of particulate grafting have prompted increasing interest in hydrogel-assisted regenerative strategies. By creating a hydrated, three-dimensional environment, hydrogels support cell survival, migration, and the transport of nutrients and signaling molecules, making them well-suited for regenerative applications in surgical settings [26,27,28]. Their principal contribution, however, has stayed biological rather than mechanical. Most clinically available hydrogel-based systems provide a favorable cellular microenvironment but offer limited resistance to early physiologic loading or deformation imposed by the surrounding soft tissues [29,30,31].

Clinical translation of fully integrated injectable bone graft systems has also been influenced by a myriad of regulatory considerations. Formulations combining polymeric carriers, particulate mineral scaffolds, and biologically active agents are typically evaluated through multiple regulatory pathways, increasing the complexity of clinical development. Accordingly, currently FDA-approved or widely used injectable materials—such as recombinant growth factor–based hydrogels (e.g., GEM 21) or enamel matrix derivative formulations (e.g., Emdogain)—consist predominantly of polymeric or proteinaceous matrices that deliver biological cues, rather than composite systems embodying particulate bone grafts [32,33]. Despite their clinical utility, these materials do not provide the particulate scaffold long recognized as essential for space maintenance, osteoconduction, and volumetric stability in bone augmentation [14,15]. To date, no injectable system delivered through a true needle-orifice has successfully integrated particulate bone grafts while conserving both mechanical competence and translational practicality [24,25,34].

### 1.3. Needle-Orifice Delivery as a Mechanobiological Boundary Condition

Injection through a needle exposes injectable regenerative systems to a distinct set of physical conditions that often receive less attention than their biological composition, including visco-elastic flow, shear stress, phase-stability requirements, and an abrupt transition from extrusion to tissue confinement [35,36]. Following instillation from the needle, the construct is positioned within a mechanically constrained environment (i.e., subperiosteal pouch), most commonly beneath the periosteum or within another restricted anatomical space, where continuous compression from surrounding tissues acts on the construct before stable internal organization is established. In other words, intrinsic tissue compression, limited capacity for expansion, and resistance to displacement emerge as inherent challenges immediately after placement. These early mechanical conditions impact material homogeneity, the uniform spatial distribution of particulates and cells, and the initial mechanical state of the grafted construct—invaluable pre-requisites of successful bone tissue engineering (BTE). Otherwise, the construct enters the earliest phase of mechanotransductive signaling with an altered physical architecture, potentially influencing subsequent cell–matrix interactions [2,6,37]; in mechanically extra-permissive or unstable systems, cytoskeletal organization and focal adhesion [38] maturation are disrupted before cellular cascades can be effectively propagated, resulting in a loss of biological potential during the earliest stages of osteogenic regeneration. From a practical standpoint, the construct needs to solidify soon after extrusion to retain its shape, resist compression from surrounding tissues, and preserve the spatial organization of the mineralized microarchitecture during the early stage of healing.

These mechanical events occur while osteogenic cells are only beginning the process of woven bone formation. Consequently, the injection event fixes both where the graft sits and the mechanical conditions early osteogenesis will unfold within. This intuitive rationale is the point at which the physical behavior of the construct first begins to influence the biological response [1,3]; injectable systems that cannot preserve their internal organization throughout this transition may lose regenerative efficiency even when their composition and biochemical characteristics are otherwise favorable. For this reason, platforms should stay compositionally stable during confined maintenance and develop sufficient mechanical integrity soon after placement to maintain a uniform distribution before coagulation is further progressed. This requirement becomes especially strict when particulate mineral scaffolds are incorporated, because preserving spatial organization and developing early rigidity are key to sustaining matrix–cell interactions, resisting compressive tissue deformation, and enabling subsequent osteogenic progression.

### 1.4. Mechanotransduction and Mineral Signaling as Coupled Regulators of Osteogenesis

Cells do not respond to mechanical cues in a uniform manner. Rather, the physical properties of the extracellular matrix—including substrate stiffness, viscoelasticity, and spatial constraint—shape cytoskeletal tension, integrin engagement, and nuclear mechanotransduction, ultimately influencing osteogenic differentiation [1,2,4]. These mechanical signals regulate osteogenic gene expression and extracellular matrix deposition in mesenchymal stem cells (MSCs) [3,6]. Their biological effects depend not only on the presence of the applied stimulus, but also on when it occurs, how long it persists, and how consistently it is presented during the earliest stages of lineage commitment [31,36,39].

Calcium phosphate minerals also contribute to the early regenerative environment beyond serving as osteoconductive scaffolds [17,18,40]. Their surfaces provide sites for cellular attachment and cytoskeletal organization, while the spatial relationship among mineral particles influences local matrix maturation and subsequent mineral deposition [19] besides calcium and phosphate ions released from the mineral phase further participating in osteogenic signaling. Critically, corresponding biological response develops within the surrounding spatial and mechanical environment, in which structural instability or graft deformation may disrupt these geometric zones and, therefore, mineral-associated interactions.

Consequently, development of injectable bone graft systems has advanced both the mechanical and biological aspects of regeneration. Integration of these multi-factorial characteristics within a singular modality, however, remains limited, particularly when homogeneous delivery, spatial organization, and early structural stability must all be preserved simultaneously [25,26]. In other words, the injected construct may undergo macro-alterations in mechanical or micro-dissipation in mineral organization during the earliest stages after placement, conditions that can ultimately influence osteogenic regeneration.

Subsequent regenerative systems should therefore treat mechanical conditioning and mineral acquisition as interrelated components rather than independent design variables. This relationship becomes particularly relevant during the earliest stage of healing, when mechanotransductive signaling and osteogenic lineage commitment are first established.

### 1.5. Design Rationale and Study Objectives

**Figure 1:**
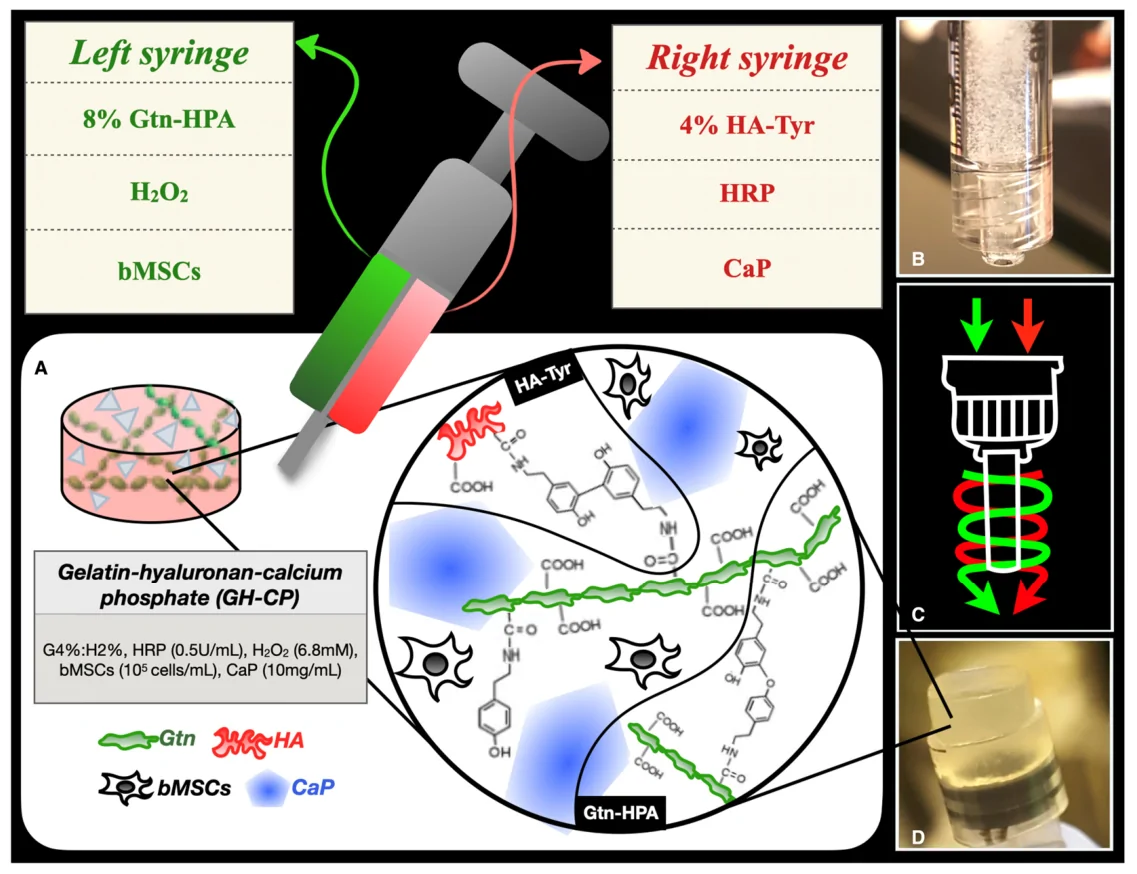
**Scheme of the proposed molecular mechanism of the gelatin–hyaluronan–calcium phosphate (GH-CP) gel cast fabrication using a needle-orifice dual-syringe injectable delivery system.** (**A**) Molecular schematic illustrating the injectable GH-CP system. Gelatin–hydroxyphenyl propionic acid (Gtn-HPA) and hyaluronic acid–tyramine (HA-Tyr) conjugates are loaded separately into a dual-syringe system containing horseradish peroxidase (HRP)/hydrogen peroxide (H_2_O_2_) cross-linking components, calcium phosphate (CaP) particulates, and bone marrow-derived mesenchymal stem cells (bMSCs). Upon mixing, HRP-mediated oxidative coupling of phenolic moieties on Gtn-HPA and HA-Tyr initiates rapid covalent cross-linking, producing a three-dimensional hydrogel network that remains injectable during extrusion yet rapidly acquires structural integrity following delivery under physiological conditions. (**B**) Homogeneous pre-dispersion of CaP particulates within pre-gelated HA-Tyr before mixing. (**C**) Schematic illustration of the auto-mixing tip enabling controlled in situ blending of both syringe components immediately before extrusion. (**D**) Representative cross-linked three-dimensional GH-CP construct (6 mm diameter; 3–4 mm thickness) following post-extrusion gelation.

The present hydrogel–mineral platform was developed as an injectable construct in which the mechanical and biological components were considered simultaneously (Table 1). Particular attention was directed toward maintaining structural integrity during needle-orifice delivery while preserving a favorable environment for subsequent osteogenic regeneration. Gelatin- and hyaluronan-based hydrogel networks were combined with particulate calcium phosphate and configured to undergo rapid enzymatic cross-linking immediately after extrusion [24,25]. Injection is followed by gelation of the precursor into a hydrated construct that remains mechanically stable during the initial cross-linking. Throughout this process, the hydrogel matrix, calcium phosphate particles, and encapsulated cells retain a homogeneous distribution, as the construct acquires sufficient early mechanical stability to withstand physiologic compressive loading [1,31] (Figure 1).

**Table 1:** Composition of the hydrogel formulations used throughout the study, including the constituent components, polymer concentrations, enzymatic cross-linking reagents, calcium phosphate (CaP) content, goat bone-marrow derived mesenchymal stem cell (bMSC) incorporation, and corresponding hydrogel designation. bMSCs were incorporated only for cell-based biological assays, whereas acellular formulations were used for rheological, mechanical, swelling, and ex vivo experiments. All formulations were prepared by enzymatic cross-linking using horseradish peroxidase (0.5 U/mL) and hydrogen peroxide (6.8 mM). Gtn-HPA and HA-Tyr precursor solutions were prepared at 8 wt% and 4 wt%, respectively. Unless otherwise stated, formulations were mixed at a 1:1 volume ratio immediately before gelation using the in-house, custom-fabricated dual-syringe auto-mix system.

Formulation	Description	Gtn-HPA	HA-Tyr	HRP	H_2_O_2_	CaP	bMSCs
G	Gelatin hydrogel	8 wt%	n/a	0.5 U/mL	6.8 mM	n/a	Optional
H	Hyaluronan hydrogel	n/a	4 wt%	0.5 U/mL	6.8 mM	n/a	Optional
GH	Gelatin–hyaluronan hydrogel	8 wt%	4 wt%	0.5 U/mL	6.8 mM	n/a	Optional
GH-CP	Gelatin–hyaluronan–calcium phosphate composite	8 wt%	4 wt%	0.5 U/mL	6.8 mM	10 mg/mL	1 × 10^5^ cells/mL

Mechanical events immediately following injection establish the microenvironment that osteogenic cells encounter before active regeneration begins. Hence, early preservation of structural stability maintains the relationship between the surrounding matrix, mineral surfaces, and resident cells, allowing mechanotransductive signaling to develop within the initial regenerative microenvironment [2,6,19]. For this reason, injectability and early mechanical behavior were considered together throughout the development of the composite because both contribute to subsequent cellular responses and the spatial organization of BTE.

**Figure 2:**
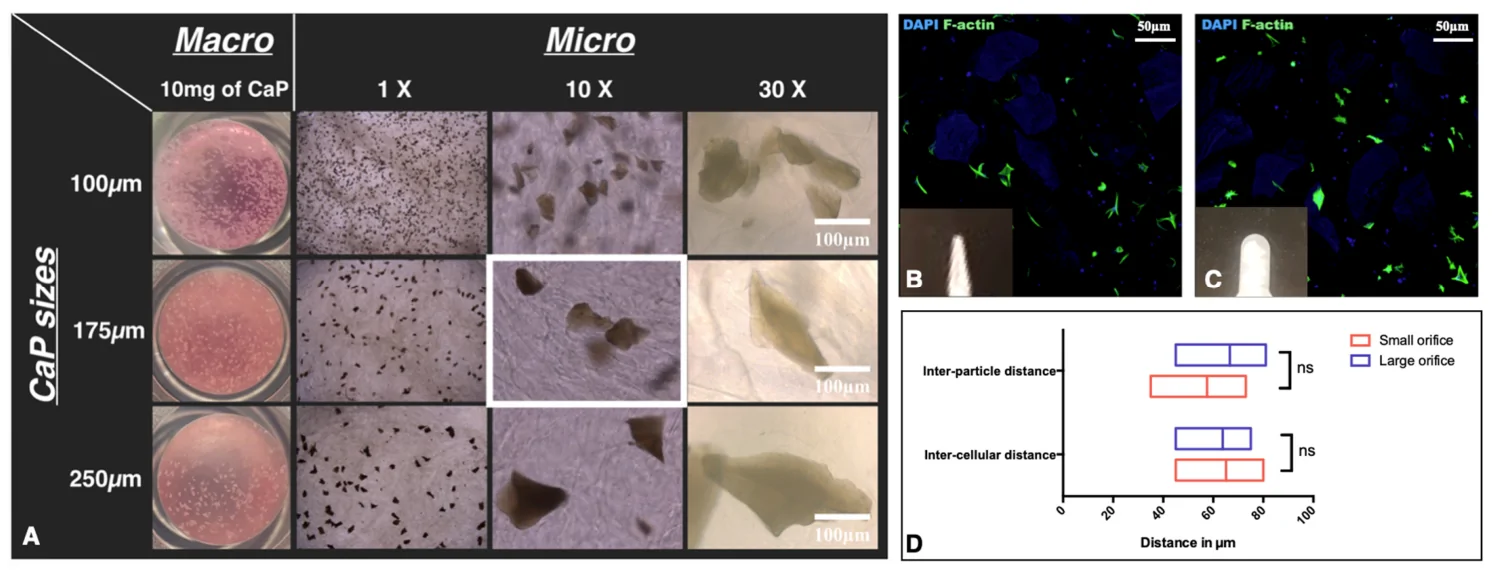
**Microscopic evaluation of fabricated GH-CP gel cast constructs.** (**A**) Representative micrographs demonstrating uniform inter-particulate spacing among CaP particles (10 mg/mL; *n* = 4). (**B**,**C**) Spatial distribution of bone marrow-derived mesenchymal stem cells (bMSCs) and CaP particulates within cross-linked GH-CP delivered through small- and large-orifice tips. Nuclei and actin cytoskeleton were visualized using DAPI and phalloidin (F-actin staining solution; Thermo Fisher Scientific, Waltham, MA, USA), respectively. (**D**) Quantitative comparison of inter-particulate and inter-cellular distances. Two-way ANOVA (*n* = 4); data are presented as mean ± minimum and maximum; ns, not statistically significant, *p* > 0.05.

**Figure 3:**
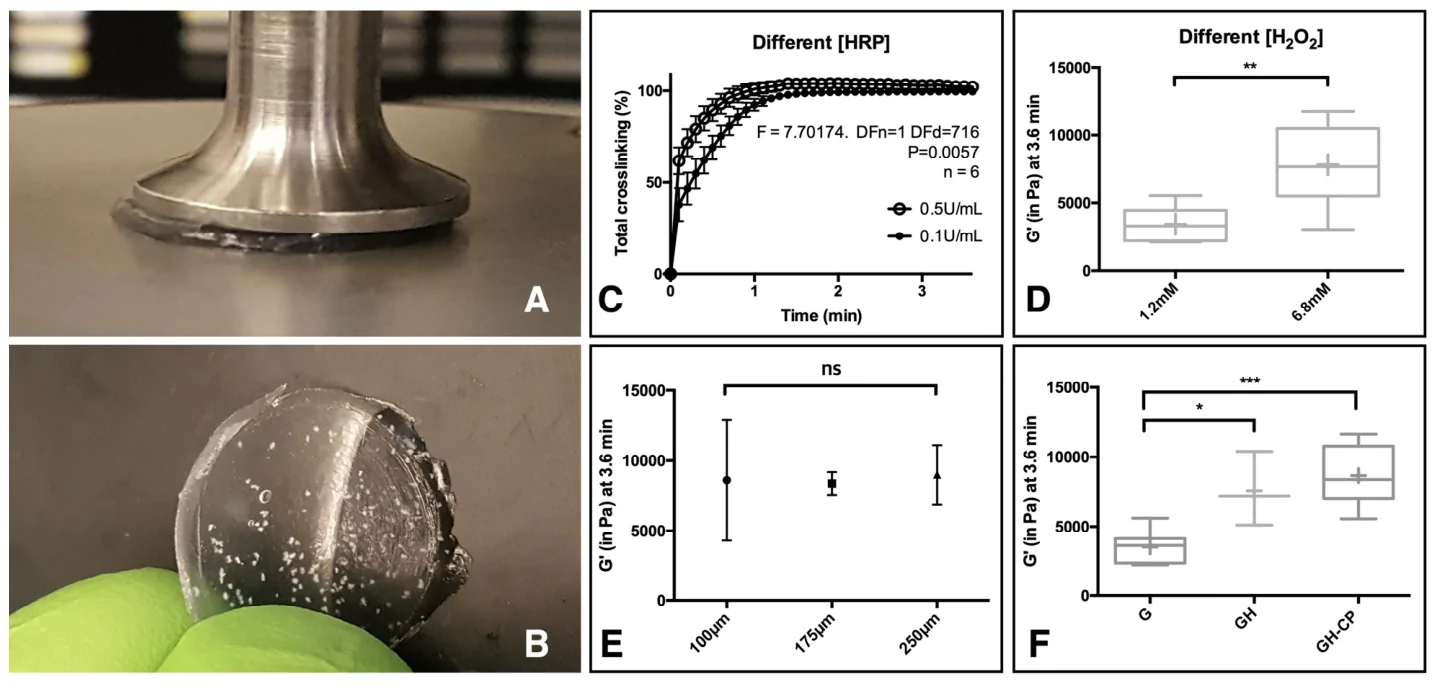
**Rheological characterization during the gelation of GH-CP using a TA Instruments AR-G2 rheometer (TA Instruments, New Castle, DE, USA).** (**A**) Parallel-plate geometry (20-mm diameter) under oscillatory compression. (**B**) Post-gelated GH-CP construct following rheological testing. (**C**) Total cross-linking efficiency as a function of horseradish peroxidase (HRP) concentration (*n* = 6; mean ± s.e.m.). (**D**) Shear storage modulus (G′) at 3.6 min as a function of hydrogen peroxide (H_2_O_2_) concentration (unpaired *t*-test; *n* = 6; box-and-whisker plot; ‘+’ denotes the mean). (**E**) Shear storage modulus (G′) at 3.6 min of GH-CP formulated with different CaP particle sizes (one-way ANOVA; *n* = 2; mean ± s.d.). (**F**) Shear storage modulus (G′) at 3.6 min of GH-CP across different gel formulations (one-way ANOVA with Tukey’s post hoc test: G (*n* = 7), GH (*n* = 3), GH-CH (*n* = 6), mean ± S.D.); box-and-whisker plot; ‘+’ denotes the mean). ns, not statistically significant, * *p* < 0.05; ** *p* < 0.01; *** *p* < 0.001.

**Figure 4:**
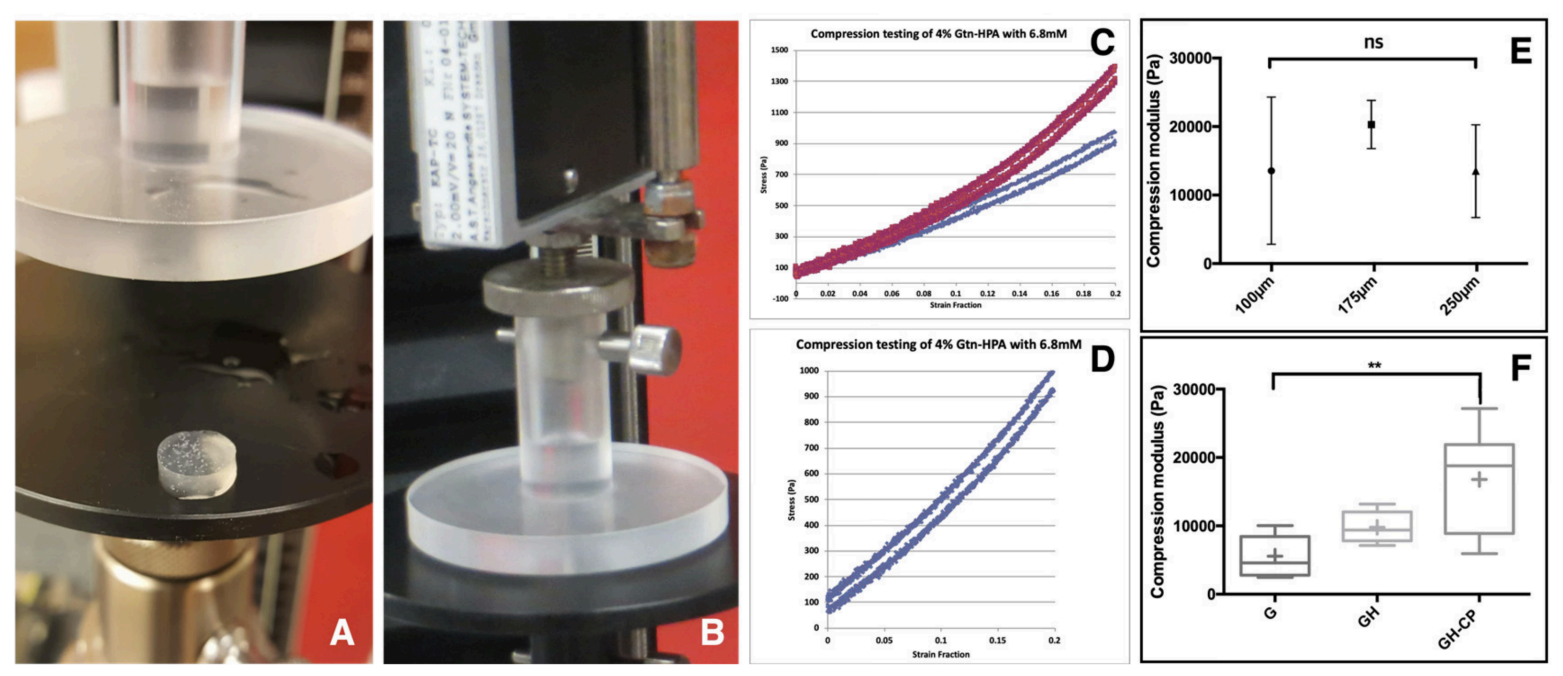
**Compressive mechanical characterization of GH-CP gel casts using a static mechanical testing system (Zwick/Roell Z2.5; Zwick GmbH & Co., Ulm, Germany).** Representative GH-CP constructs (**A**) before and (**B**) after uniaxial compression. (**C**) Representative nominal (red) and true (blue) stress–strain curves. (**D**) Representative loading–unloading response under compressive testing. (**E**) Compressive modulus (E, Pa) of GH-CP formulated with different CaP particle sizes (*n* = 2; mean ± s.d.). (**F**) Compressive modulus (E, Pa) across different GH-CP gel formulations (one-way ANOVA with Tukey’s post hoc test; G (*n* = 8), GH (*n* = 6), GH-CH (*n* = 6); box-and-whisker plot; ‘+’ denotes the mean). ns, not statistically significant, ** *p* < 0.01.

**Figure 5:**
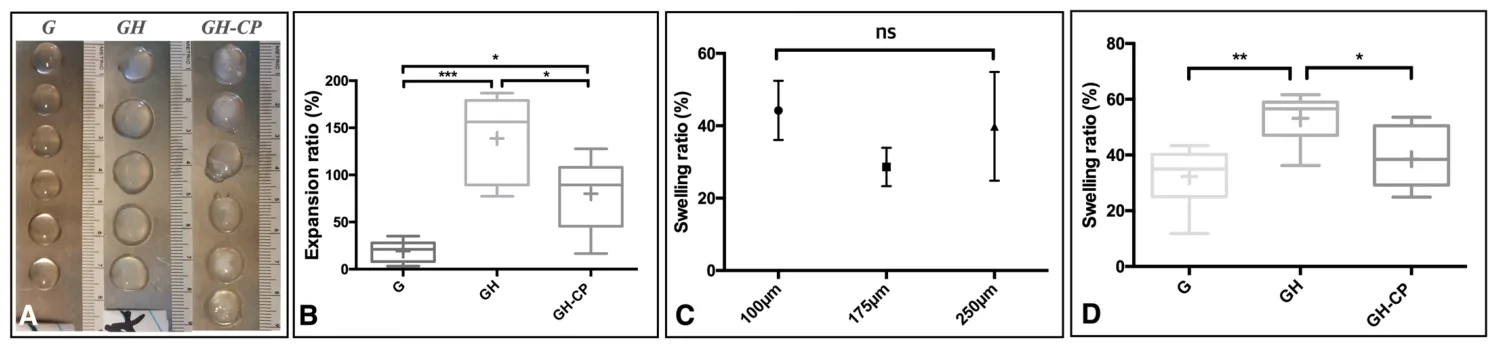
**Volumetric expansion and swelling behavior of GH-CP constructs.** (**A**) Representative gross images of gel formulations after 7 days for expansion ratio analysis. (**B**) Expansion ratio across GH-CP gel formulations (scale bar, 10 mm; one-way ANOVA with Tukey’s post hoc test: G (*n* = 6), GH (*n* = 5), GH-CH (*n* = 6), box-and-whisker plot; ‘+’ denotes the mean). (**C**) Swelling ratio as a function of CaP particle size after 7 days (*n* = 3; mean ± s.d.; ns). (**D**) Swelling ratio across GH-CP gel formulations after 7 days (one-way ANOVA with Tukey’s post hoc test; *n* = 6; box-and-whisker plot; ‘+’ denotes the mean). ns, not statistically significant, * *p* < 0.05; ** *p* < 0.01; *** *p* < 0.001.

**Figure 6:**
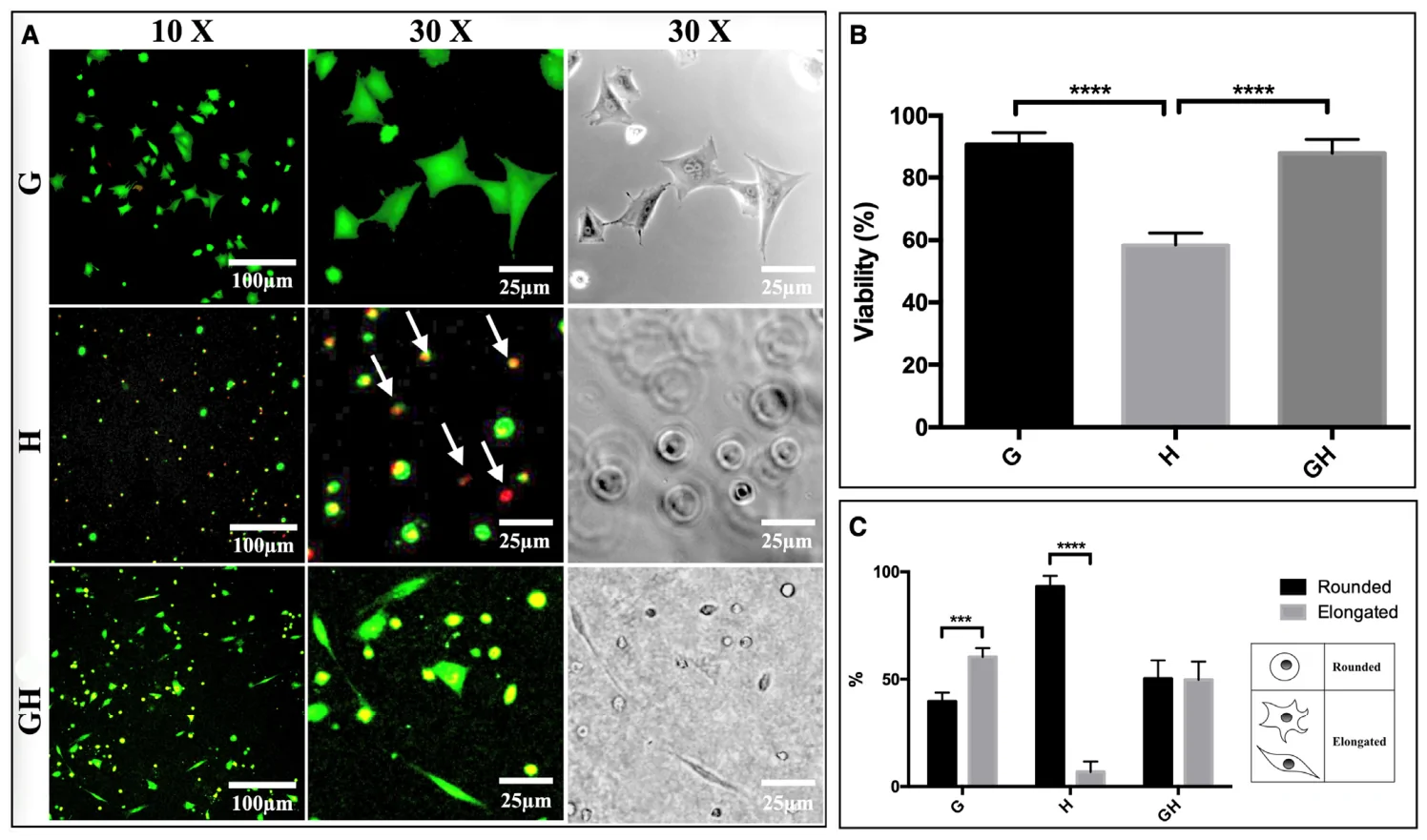
**Cytocompatibility and morphological characteristics of bMSCs across hydrogel formulations.** (**A**) Cytocompatibility assessment of bone marrow-derived mesenchymal stem cells (bMSCs) using Live/Dead Viability/Cytotoxicity Kit (Molecular Probes, Thermo Fisher Scientific, Eugene, OR, USA). Live cells were stained with Calcein AM, and dead cells with ethidium homodimer-1 (EthD-1; white arrows). Cell viability was quantified as the ratio of live cells to total cells after 24 h. (**B**) Quantitative analysis of bMSC viability across different gel formulations (one-way ANOVA; *n* = 6; mean ± s.d.; **** *p* < 0.0001). (**C**) Quantification of bMSC morphology across different gel formulations (two-way ANOVA; *n* = 6; mean ± s.e.m.; *** *p* < 0.001; **** *p* < 0.0001). All groups were cultured under identical expansion-medium conditions without osteogenic induction. Arrow signs represent dead cells.

**Figure 7:**
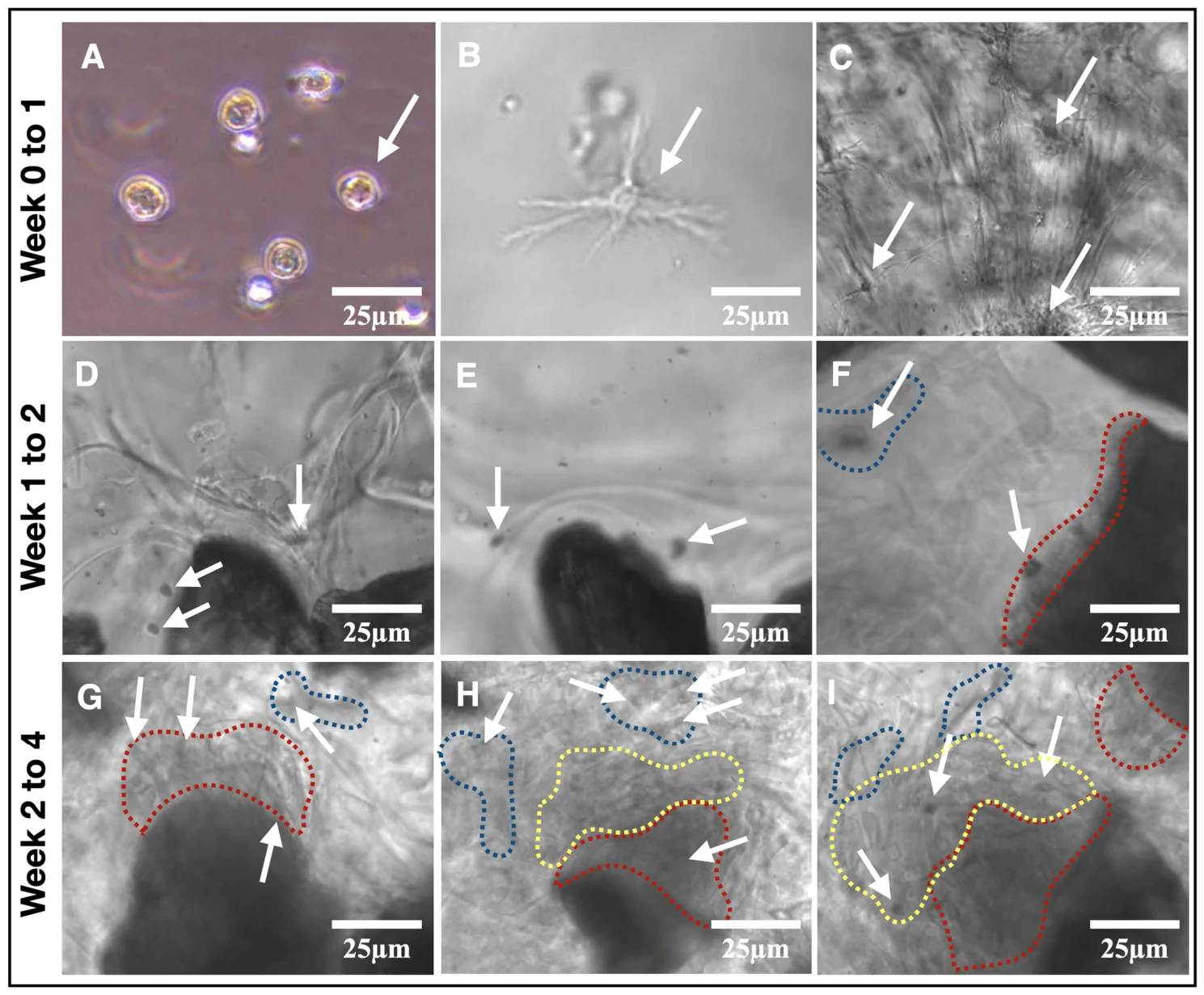
**Time-resolved microscopic observations of de novo bone formation by bone marrow-derived mesenchymal stem cells (bMSCs) within GH-CP in vitro.** (**A**) bMSCs cultured in expansion medium (week 0–1). (**B**) bMSCs cultured in osteogenic medium (week 0–1). (**C**) Formation of HIGS (see Figure 11) within GH-CP. (**D**) Initial interaction of bMSCs with the surface of CaP particles (week 1–2). (**E**) Adherent bMSCs at the CaP interface (week 1–2). (**F**) Spatially distinct regions consistent with contact osteogenesis (red) and distance osteogenesis (blue). (**G**) Early-stage de novo bone-like matrix formation (week 2–4). (**H**) Progressive maturation of bone-like tissue within GH-CP (week 2–4). (**I**) Distinct interfacial zone, delineating the osteoid/calcified-bone boundary, consistent with the proposed *cling osteogenesis* (yellow) (see Figure 11). Representative microscopic images obtained under identical culture conditions. Arrow signs represent bMSCs.

**Figure 8:**
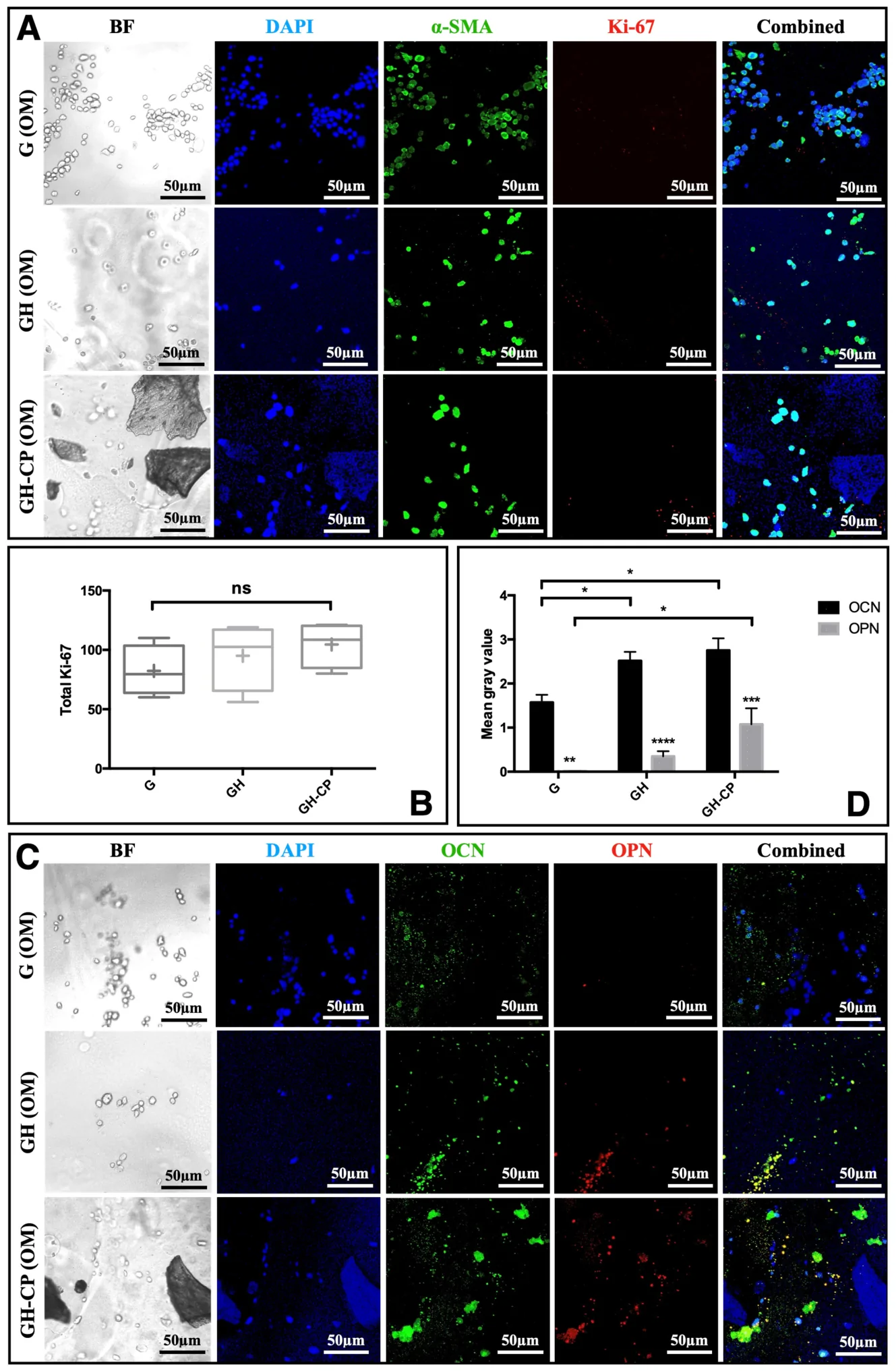
(**A**) Representative bright-field and immunofluorescence images of α-smooth muscle actin (α-SMA) and Ki-67 staining in G, GH, and GH-CP after 28 days of culture. Nuclei were counterstained with DAPI. (**B**) Quantification of Ki-67-positive cells across G, GH, and GH-CP formulations (one-way ANOVA; *n* = 6; box-and-whisker plot; “+” denotes the mean; ns, *p* > 0.05). (**C**) Representative bright-field and immunofluorescence images of osteocalcin (OCN) and osteopontin (OPN) expression in G, GH, and GH-CP after 28 days of osteogenic culture. (**D**) Quantification of OCN and OPN mean gray fluorescence intensity across G, GH, and GH-CP formulations (two-way ANOVA; *n* = 6; mean ± s.e.m.; ns, not statistically significant, * *p* < 0.05; ** *p* < 0.01; *** *p* < 0.001; **** *p* < 0.0001). Scale bars, 50 µm. Horizontal brackets indicate comparisons among hydrogel formulations; significance symbols positioned directly above individual OPN bars indicate OCN-versus-OPN comparisons within the corresponding formulation. OM indicates osteogenic medium. Negative immunostaining controls prepared using isotype IgG in place of the primary antibody are described in Section 2.

**Figure 9:**
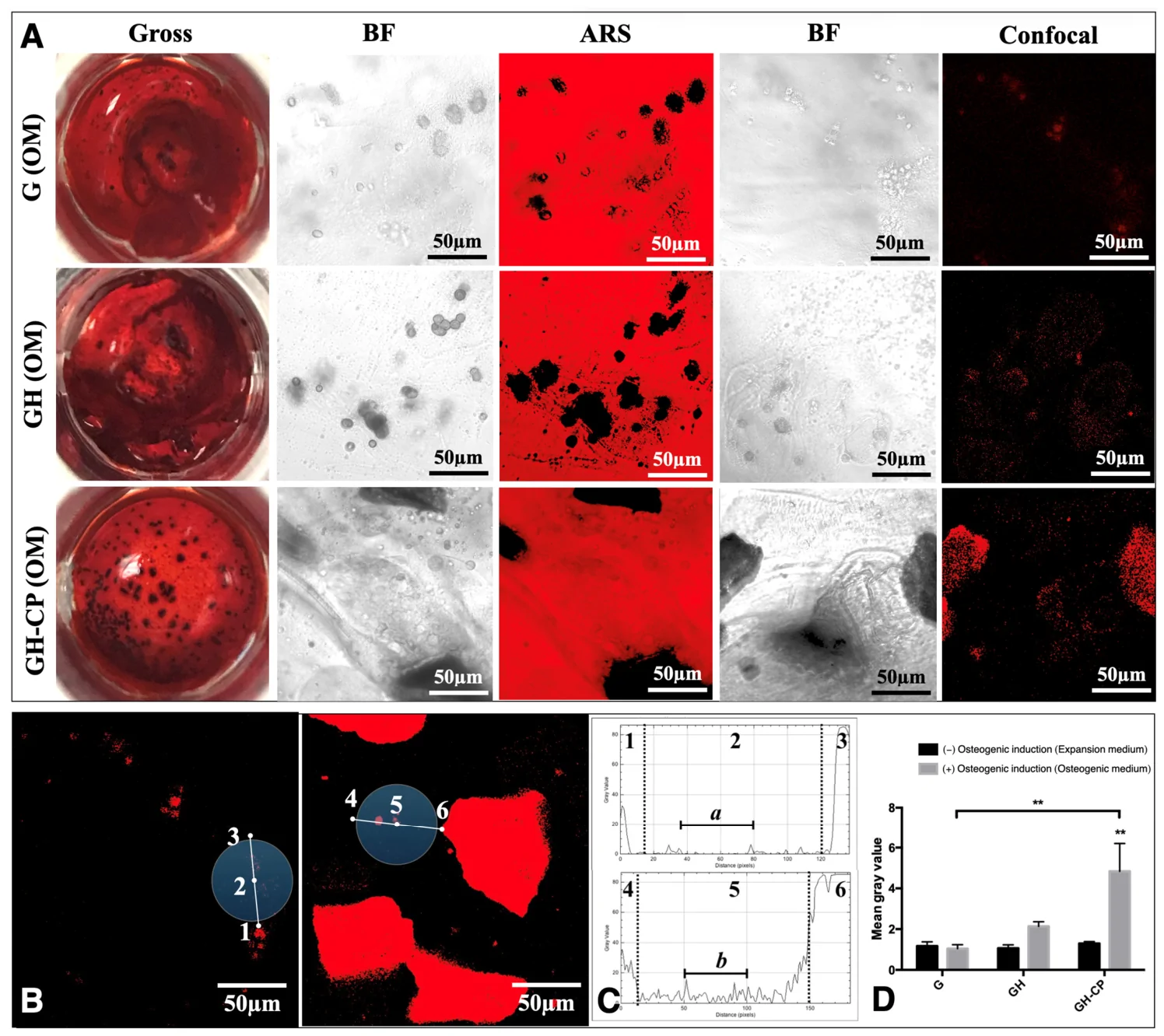
**Matrix mineralization and spatial distribution of calcium deposition within the hydrogel constructs.** (**A**) Representative Alizarin Red S (ARS) staining (Sigma-Aldrich, St. Louis, MO, USA) after 28 days of culture in G, GH, and GH-CP. Calcium deposits appear as dark nodules under bright-field microscopy and as red fluorescence under confocal microscopy. (**B**) Inter-substratum spatial profiling of ARS fluorescence surrounding a cell within GH and calcium phosphate particles within GH-CP. Circular regions of interest (100 µm diameter) were positioned at the exterior edge of visible cell-associated regions and/or pre-existing CaP particles to minimize confounding effect. (**C**) Representative ARS intensity profiles measured across the selected regions (above: GH; below: GH-CP). (**D**) Quantification of ARS mean gray fluorescence intensity across G, GH, and GH-CP under expansion (−) and osteogenic (+) culture conditions (two-way ANOVA; *n* = 6; mean ± s.e.m.; ** *p* < 0.01). Scale bars: 50 µm, unless otherwise indicated. OM indicates osteogenic medium.

**Figure 10:**
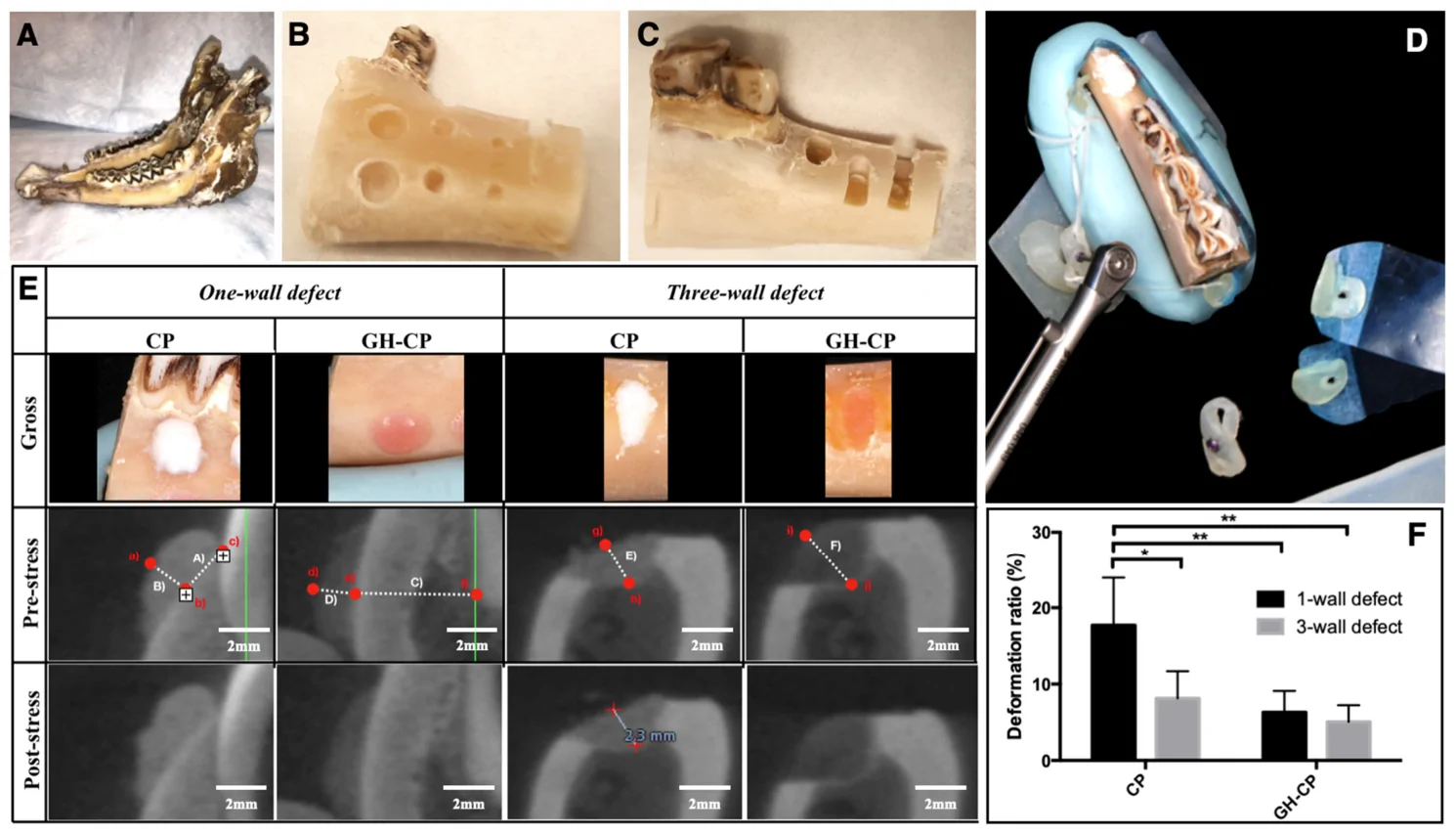
**Ex vivo bony defect model and volumetric deformation assessment under controlled mechanical constraint.** (**A**) Surgically extracted goat mandible used as an ex vivo bony tissue model. (**B**) Circular corticotomies of varying diameters generated within cortical bone for calibration. (**C**) Development of bony defect configurations with different defect wall geometries. (**D**) Assembled mucoperiosteal flap tension system applied to impose a consistent compressive constraint on bony defect sites, mimicking periosteal containment of grafted regions. (**E**) Representative CBCT volumetric deformation images obtained from one-wall and three-wall bony defect models. (**F**) Quantitative deformation ratio (%) comparing defect configurations (two-way ANOVA with Tukey’s post hoc test; *n* = 4; mean ± s.d.; * *p* < 0.05; ** *p* < 0.01).

The study was designed to follow the sequence of investigations that an injectable graft experiences under surgically relevant delivery conditions through: (i) needle-orifice extrusion (Figure 1 and Figure 2); (ii) rheological behavior before and after gelation (Figure 3); (iii) compressive mechanical properties (Figure 4); (iv) volumetric expansion in a body-fluid-mimicking environment (Figure 5); (v) cellular viability (Figure 6); (vi) cytoskeletal organization (Figure 7); (vii) osteogenic differentiation (Figure 8); (viii) spatial mineralization (Figure 9); (ix) volumetric stability in bony defect models (Figure 10). Rather than examining these properties independently, the aim was to navigate how early mechanical stabilization of the hydrogel–mineral construct and its unique geometric micro-architectures influence subsequent osteogenic sequelae, within optically discoverable hydrogel intersubstratum and anatomically mimicked, mechanically demanding regenerative environments.

## 2. Materials and Methods

### 2.1. Materials

Gelatin–hydroxyphenyl propionic acid conjugate (Gtn–HPA) and hyaluronic acid–tyramine conjugate (HA–Tyr) were provided by the Institute of Bioengineering and Nanotechnology (IBN, A*STAR, Singapore, Singapore) as previously synthesized conjugates. Gtn–HPA was produced via carbodiimide/active ester-mediated coupling reactions as previously reported [41,42]. The parent gelatin used for conjugation had a molecular weight of 80–140 kDa and an isoelectric point (pI) of approximately 5. The degree of substitution, defined as the percentage of gelatin primary amine groups conjugated with hydroxyphenyl propionic acid, was approximately 90%, as determined by the 2,4,6-trinitrobenzene sulfonic acid (TNBS) assay [43].

Hyaluronic acid–tyramine conjugate (HA–Tyr), previously synthesized and characterized by the same institution, was provided as a pre-synthesized conjugate. The degree of substitution was approximately six tyramine moieties per 100 repeating units of hyaluronic acid, as confirmed by 1H nuclear magnetic resonance (NMR) spectroscopy [44,45].

Horseradish peroxidase (HRP; 25 U mL^−1^ stock solution) was purchased from Wako Chemicals USA, Inc. (Richmond, VA, USA). Hydrogen peroxide (H_2_O_2_; 9.7 M stock solution) was purchased from Sigma-Aldrich (St. Louis, MO, USA). Pre-filtered calcium phosphate (CaP) particles consisting of deproteinized bovine bone mineral (DBBM), composed predominantly of naturally derived hydroxyapatite (HA), with nominal diameters of approximately 100 μm, 175 μm, and 250 μm, were supplied by Geistlich Pharma (Wolhusen, Switzerland).

A CaP concentration of 10 mg/mL maintained injectability through the dual-syringe delivery system while reducing particle sedimentation and nozzle clogging during extrusion (Figures S1 and S2). An intermediate particle size (~175 μm) preserved sufficient intersubstratum spacing for microscopic characterization of molecular biological investigation (Figure 2). This combination ultimately enabled detailed evaluation of the hydrogel–mineral interface (Table 2).

Phosphate-buffered saline (PBS), α-minimum essential medium (αMEM), fetal bovine serum (FBS), trypsin–EDTA, and antibiotic–antimycotic solution were obtained from Gibco (Thermo Fisher Scientific, Waltham, MA, USA). All reagents were used as received unless otherwise stated.

### 2.2. Design and Fabrication of the Dual-Syringe Auto-Mix Injectable System

A custom dual-barrel syringe with an auto-mix tip was prepared for all injections. The HA–Tyr pre-gel solution containing CaP particles and HRP was loaded into one barrel, whereas the Gtn–HPA solution containing H_2_O_2_ was placed in the other. Because the two precursor solutions remained physically separated until injection, no visible gelation occurred during syringe loading or handling (Figure 1 and Figure S2).

Before use, both precursor solutions were kept at 37 °C for 1 h. This step was included primarily to obtain a consistent viscosity while minimizing sedimentation of the CaP particles before injection. The two solutions first came into contact inside the auto-mix tip and were immediately delivered through the needle-shaped orifice. Under these conditions, extrusion remained smooth, with no evidence of clogging, phase separation, or uneven particle distribution (Figure 2).

The hydrogel formulations evaluated in this study consisted of gelatin–hydroxyphenyl propionic acid (G), hyaluronic acid–tyramine (H), gelatin–hyaluronan (GH), and gelatin–hyaluronan containing calcium phosphate particles (GH-CP). These groups were designed to distinguish the individual and combined contributions of gelatin, hyaluronic acid, and CaP particles throughout the study (Table 1).

### 2.3. Preparation of Enzymatically Cross-Linked Hydrogels

#### 2.3.1. Gtn–HPA Hydrogel

Gtn–HPA conjugates were dissolved in PBS to a final concentration of 8 wt% (degree of substitution in Section 2.1).

#### 2.3.2. HA–Tyr Hydrogel

HA–Tyr conjugates were dissolved in PBS to a final concentration of 4 wt% (degree of substitution in Section 2.1)

#### 2.3.3. Enzymatic Covalent Cross-Linking

The precursor solutions were mixed within the static auto-mix tip during extrusion, initiating rapid HRP-mediated oxidative coupling of the phenolic groups under physiological conditions. Subsequent casting procedures depended on the experimental protocol and are described in Section 2.5. All preparations were performed aseptically at room temperature (approximately 21 °C) unless otherwise specified.

### 2.4. Incorporation of CaP Particles

Calcium phosphate (CaP) particles were incorporated into the HA–Tyr precursor solution before loading into the corresponding barrel of the dual-syringe system. Preliminary optimization studies additionally confirmed that this concentration preserved mechanical integrity and cellular compatibility, supporting its use throughout the study [46].

### 2.5. Direct and Indirect Gel Casting and Sterilization

Following extrusion from the dual-syringe auto-mix system described above, the freshly mixed hydrogel precursor was processed using one of the following casting methods, as specified in the experimental protocol. Hydrogels were prepared using either direct or indirect gel casting, depending on the subsequent experiment. Direct gel casting was used for cell-based biological assays, including cell viability, morphology, immunofluorescence, osteogenic differentiation, mineralization analyses, and ex vivo experiment (Figure 6, Figure 7, Figure 8, Figure 9 and Figure 10). Indirect gel casting was used to prepare free-standing cylindrical constructs for the initial optimization, compression testing and swelling analyses (Figure 2, Figure 3, Figure 4 and Figure 5).

Before gel preparation, the syringe barrels, static mixing tips, syringe-section molds, and CaP particles were sterilized under ultraviolet (UV) light in a laminar flow hood for 7 days. Disposable syringe barrels were then cut into short cylindrical sections to serve as temporary molds for indirect gel casting. The molds were further rinsed with 75% ethanol, washed thoroughly with sterile deionized water, allowed to air dry, and sealed at one end with a sterile parafilm square (approximately 1.5 × 1.5 cm).

For indirect gel casting, 250 μL of the freshly mixed hydrogel precursor was dispensed directly into each cylindrical mold immediately after dual-syringe extrusion. Gelation proceeded at room temperature (21 °C), after which the free-standing cylindrical constructs were carefully removed from the molds (Figure 1) and used for compression testing and swelling analyses.

For direct gel casting, mixed precursor solutions were dispensed directly into sterile culture dish wells using the dual-syringe system to produce hydrogel constructs (120 μL per sample). Cell-laden constructs were prepared immediately after extrusion under sterile conditions. All procedures involving cell-containing hydrogels were performed aseptically in a biosafety cabinet at approximately 21 °C, followed by incubation at 37 °C until cross-linking was complete, prior to subsequent biological analyses.

### 2.6. Isolation and Culture of Bone Marrow-Derived Mesenchymal Stem Cells (bMSCs)

Bone marrow aspirates were obtained from the iliac crest of adult female Spanish goats (2–3 years old; approximately 45 kg). The cells were derived from tissues collected from an animal carcass after the animal had died from causes unrelated to the present study. Aspirates were collected into sterile tubes containing cold PBS supplemented with 1 mM ethylenediaminetetraacetic acid (EDTA). Cell isolation was performed following the protocol originally described by Friedenstein et al. [47]. The isolation, expansion, and biological characterization of goat bMSCs obtained using this protocol have been previously established by our laboratory through immunophenotypic characterization (CD29, CD44, CD105) together with functional osteogenic differentiation assays [48,49].

Isolated cells were cultured in αMEM supplemented with 10% FBS and antibiotic–antimycotic solution at 37 °C in a humidified incubator with 5% CO_2_. Cells were passaged upon reaching confluence using trypsin–EDTA, centrifuged at 1500 rpm for 10 min, and resuspended for counting using a hemocytometer with trypan blue exclusion. Cells were encapsulated at a final density of 1 × 10^5^ cells mL^−1^.

Goat bMSCs were selected to maintain biological consistency with the caprine tissues and ex vivo models used throughout this investigation, while supporting the planned translational progression toward an in vivo caprine guided bone regeneration model. Human bMSCs were therefore not employed, as the primary objective of this study was to evaluate the hydrogel–mineral system within a single preclinical animal model rather than to compare species-specific cellular responses.

### 2.7. Rheological Characterization

The rheological method was utilized to analyze viscoelastic status during gelation. Immediately after extrusion through the dual-syringe system, mixed precursor solutions were transferred onto the rheometer stage for oscillatory measurements. Rheological measurements were obtained using an AR-G2 rheometer (TA Instruments, New Castle, DE, USA) fitted with a 20 mm parallel-plate geometry (Figure 3A,B). Samples were analyzed at 37 °C under oscillatory conditions with 1% strain and 1 Hz, which remained within the linear viscoelastic range. To minimize dehydration during testing, a thin layer of silicone oil was placed around the edge of each sample. An oscillation rheometer was used to measure the storage (G′), indicating the stiffness of the material, and loss moduli (G″) based on shear strain, to investigate the dynamic cross-linking of G, GH, and GH-CP gels. Additionally, different CaP particles (~250 µm, ~175 µm, and ~100 µm) with GH gel were tested as a pilot experiment. Typically, a crossover point between G′ and G″ helps estimate gelation rate. However, in this experiment, due to high [HRP], gelation occurred immediately while going through the dual-syringe tip; thereby, the crossover was already passed at the start of rheometer measurements (Figure 3C).

### 2.8. Unconfined Compression Testing

Cylindrical syringe-section molds prepared as described in Section 2.5 were filled with freshly mixed hydrogel precursor immediately after extrusion from the dual-syringe system (Figure 4A). Following gelation, the constructs were equilibrated in PBS at 37 °C for 1 h before compression testing. Specimen diameter was verified using digital calipers immediately before testing. Unconfined compression testing was performed using a Zwick/Roell Z2.5 static materials testing system (Zwick GmbH & Co., Ulm, Germany) controlled by testXpert II software, version 3.4 (Zwick GmbH & Co. KG, Ulm, Germany) (Figure 4B). Gel samples (6 mm diameter; 3–4 mm thickness) were compressed at a constant strain rate of 0.5% s^−1^ up to 10% strain, followed by unloading to zero strain. A 20 N load cell was used, and data were collected at 2 Hz. The compressive modulus was calculated from the slope of the linear region of the true stress–strain curve.

### 2.9. Swelling Ratio

Indirect gel casts (250 μL, 8–9 mm diameter, 3–4 mm height) were immersed in PBS and stored for 7 days at 37 °C to allow swelling. Tweezers were used to gently scoop the gels, protecting their structure. The weights of the gels before and after swelling were recorded as *W*_0_ and *W*_1_. The swelling ratio was calculated with the following equation:

*Swelling Ratio* = |*W*_0_ − *W*_1_|/*W*_0_ × 100%

### 2.10. Expansion Ratio

A volume of 250 μL of gel was dispensed into the bottom of cryotubes to ensure a consistent surrounding structural frame across different experimental groups (Figure 5A). Then, 500 μL of PBS was added on top of the cross-linked gels and left for 7 days at 37 °C to maximize water absorption. The gels naturally took on a hemispherical shape due to the cryotube’s design. An impression of vinyl polysiloxane (VPS) was first made to serve as a reference volume (*E*_0_). Afterward, the remaining gels were carefully removed from each cryotube, and their diameters were measured to calculate the expanded volume (*E*_1_). The hemisphere volume and expansion ratio were then determined using the following equation.

*Hemisphere volume* (*E*) = (2/3) *π r*^3^

*Expansion ratio* = *E*_1_/*E*_0_ × 100%

### 2.11. Osteogenic Differentiation

Cell-laden hydrogel constructs were maintained in expansion medium for the first 5 days of culture, and then transferred to osteogenic differentiation medium for an additional 23 days, yielding a total culture period of 28 days. Control constructs were cultured in expansion medium throughout the same interval. The culture medium was refreshed every 3 days. Osteogenic differentiation at day 28 was assessed by immunocytochemistry, immunofluorescence, and Alizarin Red S staining.

### 2.12. Immunocytochemistry and Immunofluorescence

Immunocytochemistry and immunofluorescence were used to examine osteogenic differentiation, proliferation, and cellular contractile behavior within the hydrogel constructs after 28 days of culture. At the end of the culture period, the medium was removed and each gel was fixed in 500 µL of 4% paraformaldehyde for 2 h at room temperature. The specimens were washed with 500 µL of phosphate-buffered saline (PBS) and then permeabilized in 300 µL of 0.1% Triton X-100 (Sigma-Aldrich, St. Louis, MO, USA) for 1 h at 4 °C. After another PBS wash, endogenous peroxidase activity was quenched using 5–6 drops of peroxidase-blocking reagent from the EnVision + System-HRP (AEC) kit (Dako, Carpinteria, CA, USA). This step was carried out overnight at 21 °C. Nonspecific antibody binding was subsequently reduced using serum-free protein block (Dako).

The primary antibodies were mouse monoclonal anti-osteocalcin (OCN; Abcam, Cambridge, UK; ab13418; 1:200), rabbit monoclonal anti-osteopontin (OPN; Abcam; ab8448; 1:1000), mouse monoclonal anti-α-smooth muscle actin (α-SMA; Abcam; ab5694; 1:400), and rabbit monoclonal anti-Ki-67 (Abcam; ab15580; 1:1000). Gel constructs were incubated with the corresponding primary antibodies overnight at 4 °C. Negative controls were prepared by replacing the primary antibody with a stock negative-control mouse IgG fraction (Dako) at the corresponding dilution.

After primary-antibody incubation, the constructs were washed twice in 500 µL of PBS for 2 h per wash. A further 500 µL PBS wash was then applied, and the specimens were kept overnight at 4 °C while protected from light. The PBS was removed the next day, and 250 µL of combined Alexa Fluor 488- and Cy3-conjugated secondary detection antibodies, each diluted 1:300, was added. Secondary-antibody incubation was performed overnight at 4 °C in the dark. The constructs were then washed once with 0.05% Tween 20 and once with PBS, each for 2 h. DAPI was used for nuclear counterstaining and stored for 8 h at 4 °C protected from light. Immediately before imaging, the DAPI solution was removed and replaced with PBS.

Fluorescence images were acquired using a Fluoview FV3000 confocal laser-scanning microscope (Olympus Corporation, Tokyo, Japan). The imaging settings for specimens in the same staining experiment were kept constant to enable comparison among groups. These settings included laser power, detector gain, exposure conditions, objective magnification, and the remaining acquisition parameters.

Image analysis was carried out using ImageJ software (version 1.51; National Institutes of Health, Bethesda, MD, USA). Ki-67 and OCN/OPN were analyzed differently because of their distinct staining patterns. Ki-67 appeared as discrete punctate nuclear signals, approximately 3–5 µm in diameter, and was therefore quantified by directly counting Ki-67-positive regions or discrete positive nuclear signals within the analyzed images (Figure 8A). The original analysis recorded the absolute Ki-67-positive count rather than calculating the percentage of positive cells relative to the total number of DAPI-stained nuclei (*n* = 6).

OCN and OPN staining was quantified using mean gray fluorescence intensity (Figure 8B). Four microscopic fields were selected from each gel specimen. Within each field, four circular regions of interest, each 100 µm in diameter, were placed in the areas showing the most readily detectable fluorescence. Because sparse background signal elsewhere would dilute the intensity of actively mineralizing or marker-positive foci, ROI placement was intentionally targeted to regions of detectable signal rather than randomized across the field; this approach characterizes the intensity of active deposition sites but does not represent an unbiased sampling of total construct area. Mean gray values were measured in ImageJ and averaged to obtain a representative value for each specimen. The specimen-level values were then used to compare the experimental groups (*n* = 6). α-SMA staining was evaluated to examine cytoskeletal and contractile organization within the constructs; its spatial correspondence with DAPI-stained nuclei and cellular morphology was assessed microscopically (Figure 8A).

### 2.13. Alizarin Red S Mineralization Analysis

Alizarin Red S (ARS) staining was carried out after 28 days of culture to examine mineral deposition within the cell-laden hydrogel constructs. At the completion of the culture period, the medium was removed and each gel was fixed in 500 μL of 4% paraformaldehyde for 2 h at room temperature. The constructs were then rinsed with 500 μL phosphate-buffered saline (PBS), followed by the addition of 300 μL Alizarin Red S solution (Sigma-Aldrich, St. Louis, MO, USA). After 30 min of staining, the excess dye was removed with PBS washes.

Residual Alizarin Red S remained within the hydrogel matrix after staining. The constructs were therefore maintained in PBS, with the solution replaced daily for approximately 3 weeks until excess background staining had diminished. The gels were subsequently examined under both optical and confocal fluorescence microscopy (Figure 9). Optical microscopy was used to visualize mineral deposition, whereas confocal microscopy was used to examine its dimensional distribution within the hydrogel.

ImageJ software (National Institutes of Health, Bethesda, MD, USA) was used for quantitative image analysis. Four representative microscopic fields were analyzed from each gel specimen. Within each field, four circular regions of interest (100 μm diameter) were positioned around visibly mineralized cell-associated regions while avoiding direct measurement of the incorporated calcium phosphate particles, since these represented the original scaffold rather than newly deposited mineral (Figure 9B). As with the OCN/OPN analysis (Section 2.12), ROI placement targeted regions of detectable mineralization rather than randomized fields, and therefore reflects the intensity of active mineral deposition, not an unbiased average across the whole construct. The mean gray fluorescence value was measured for each region of interest, and the remaining measurements were averaged to obtain a single representative value for each specimen prior to statistical analysis (*n* = 6).

Confocal images were further examined to assess the spatial relationship among Alizarin Red S staining, surrounding cells, and calcium phosphate particles. This evaluation distinguished mineral-associated fluorescence distributed throughout the hydrogel matrix from fluorescence corresponding to the pre-existing calcium phosphate particles (Figure 9B).

### 2.14. Ex Vivo Dimensional Cone Beam Computed Tomography (CBCT) Analysis for Its Surgical Feasibility in Guided Bone Regeneration (GBR) Environment

An ex vivo compression model was used to compare the behavior of particulate CaP particles (CP) and hydrogel–mineral composites (GH-CP) under defect-mimicking loading conditions (Figure 10A–C), as opposed to evaluating in vivo defect healing.

#### Surgically Extracted Pieces of Goat Mandibular Bone

A 2–3-year-old adult female Spanish goat (45 kg) was euthanized as part of another IRB-approved animal study in our lab, and the entire mandible was harvested through surgical dissection (Figure 10A). The mandible bone was then cut into multiple pieces and soaked in 10% buffered formalin for ten days. All defect specimens used in the deformation analysis (Section 3.4) were derived from this single donor mandible; regional variation in bone density or quality within one animal, rather than inter-animal variability, should be considered when interpreting the reported deformation values. After fully dehydrating in a fume hood for seven days, various sizes and types of GBR-mimicking defects (Figure 10B,C) were created for pilot testing and optimization.

#### CBCT Calibration of Position and Density

The sectioned mandibular pieces with specific defect sizes and locations for in vitro biomechanical testing were optimized using the Kodak 9000 Extraoral Imaging System CBCT (Carestream Health, Inc., Rochester, NY, USA). This process involved locating a consistent fixation jig position along the ideal vertical and horizontal axes and selecting appropriate voxel sizes within the software to ensure clear visualization of CP and GH-CP groups. After CBCT imaging, the Hounsfield units were properly segmented to highlight our region of interest. Subsequently, alignment calibration was performed in the CoDiagnostiX^TM^ software (version 9.7; Dental Wings GmbH, Chemnitz, Germany) to accurately display the cross-sectional images.

#### Mucoperiosteal Flap Tension (MFT) Device

A mucoperiosteal flap tension device was developed (Figure 10D) to mimic a physiological tension (i.e., ~0.15–0.2 N) [50] under guided bone regeneration surgical condition.

### 2.15. Statistical Analysis

Quantitative data are presented as mean ± standard deviation (SD) or mean ± standard error (SE), as demonstrated in figure legends. Statistical analyses were performed using GraphPad Prism 7 (GraphPad Software, San Diego, CA, USA). Comparisons between two groups were performed using unpaired *t*-tests, whereas comparisons among multiple groups were performed using one-way or two-way analysis of variance (ANOVA), followed by Tukey’s or Bonferroni–Šidák multiple-comparison testing, as appropriate. Image acquisition and quantification were not performed under blinded conditions with respect to group identity.

## 3. Results

### 3.1. Morphological and Physicochemical Characterization of the Hydrogel–CaP Composite During Needle-Orifice Delivery

#### 3.1.1. Immediate Gelation of GH-CP with a Uniform Distribution of CaP Particles and bMSCs

Whether the hydrogel–mineral system could be delivered through a needle orifice without loss of internal organization was the first objective addressed. Enzymatic cross-linking was initiated only after the two precursor solutions entered the static auto-mix tip immediately before needle-orifice extrusion (Figure 1A,C). The gelatin-only (G) formulation, however, showed visible sedimentation and phase separation before gelation (Figures S1 and S3), with either injection obstruction or non-uniform internal organization (Figure S2). In contrast, adding hyaluronic acid (H) to the pre-gel formulation helped stabilize the suspension phase (Figure 1B) and enabled CaP-containing hydrogels to pass through the needle orifice without clogging or obvious particle segregation (Figure 1D).

CaP particles remained evenly dispersed across the gel volume (Figure 2A), while a minor redistribution was detected during the subsequent culture medium period (Figure 8 and Figure 9). Optical microscopy also demonstrated a similarly uniform pattern of bMSCs (Figure 2B,C) throughout the hydrogel matrix, with no evident aggregation or separation following the immediate gelation through dual-syringe injection, yet minor cellular clustering was often observed after the biologic culturing period, particularly in the G group (elaborated further in discussion). The composite maintained continuous hydrogel–mineral integration while quantitatively preserving uniform interparticle spacing and cellular distribution across the construct (Figure 2D). Microscopic views at magnifications of 1×, 10×, and 30× were used to visualize and compare the gap distances between CaP particles (Figure 2A). All CaP particle sizes (~250 µm, ~175 µm, and ~100 µm) could be dispensed from a 1 mL pipette tip, followed by rapid in situ cross-linking (Figure S1).

Different tip sizes of the dual-syringe auto-mix model (small orifice diameter = 900 µm; large orifice diameter = 2 mm) with 175 µm CaP particles and bMSCs were tested to justify its ideal inter-particle and inter-cellular distances of cross-linked GH-CP. The results showed no statistically significant differences (*n* = 4, *p* > 0.05). Two-factor ANOVA indicated that the orifice size of auto-mix tips (small vs. large) did not significantly affect the distribution of contents within the gel, such as CaP particles and bMSCs (*p* = 0.3194). The inter-cellular distances and inter-particle distances measured through a small orifice auto-mix tip were 65.2 ± 11.6 µm and 57.5 ± 14.3 µm (*n* = 4, mean ± S.D.), respectively. For the large orifice tip, these distances were 63.8 ± 10.4 µm and 66.7 ± 13.6 µm, respectively (*n* = 4, mean ± S.D.) (Figure 2D).

#### 3.1.2. Rheological Behavior

To customize a surgically favorable injectable module (i.e., immediate gelation and rigid stiffness) for the objective of this study and clinical implication, HRP and H_2_O_2_ were incrementally fine-tuned with multiple initial pilot experiments (Figure S2). Increasing [HRP] from 0.1 U/mL to 0.5 U/mL steepened the cross-linking curve, indicating a faster gelation rate (Figure 3C) for its clinical practicality. A linear regression model showed that the increase in gelation rate was statistically significant (*p* = 0.0057). The primary rheological metric was G’ at 3.6 min, which reached the plateau of completed cross-linking % (Figure 3C). Increasing [H_2_O_2_] from 1.2 mM to 6.8 mM resulted in approximately a two-fold increase in G’ at 3.6 min (*n* = 6, *p* = 0.0047), suggesting a higher cross-linking density (Figure 3D).

During the injection, G′ already surpassed G″, signifying an immediate transition from a flowable precursor to a mechanically stabilized, cross-linked gel (Figure 3C). Within the tested conditions, one-way ANOVA detected no statistically significant effect of CaP particle size on G′ at 3.6 min (*p* = 0.9746). G’ values at 3.6 min for gelatin gels with CaP sizes of 100 µm, 175 µm, and 250 µm were 8607 ± 4304 Pa, 8369 ± 816 Pa, and 9002 ± 2118 Pa, respectively (*n* = 2, mean ± S.D.) (Figure 3E). An increase in G’ at 3.6 min was also observed across different gel groups (G, GH, and GH-CP), with statistical significance confirmed by one-way ANOVA (*p* = 0.0008). Tukey’s post hoc test revealed significant differences between G and GH (*p* < 0.05), and G and GH-CP (*p* < 0.001). The average G’ at 3.6 min was 3521 ± 1162 Pa for G, 7569 ± 2662 Pa for GH, and 8659 ± 2195 Pa for GH-CP: G (*n* = 7), GH (*n* = 3), GH-CH (*n* = 6), mean ± S.D. (Figure 3F). Overall, these physicochemical findings demonstrate rapid post-extrusion gelation and establishment of a mechanically competent hydrogel network. Gelatin–hyaluronan combination (GH) drove the principal rise in storage modulus, and CaP incorporation sustained this viscoelastic gain.

#### 3.1.3. Unconfined Compression Testing

None of the formulations showed obvious structural failure during the unconfined compression testing (Figure 4); across the physiologically relevant strain range, deformation remained limited, and physical integrity was maintained in all groups. The measured compressive moduli were also similar among formulations of different particle sizes (Figure 4E). Passage through the needle-shaped orifice, then, produced no detectable loss of bulk mechanical performance; injectability and early mechanical competence were both retained after gelation.

All gel groups exhibited both elastic and plastic behaviors, as shown by the differences between the nominal and true stress–strain curves (Figure 4C). During compression, a roughly 5% diversion was observed between these curves, indicating that the hydrogels contain both elastic and plastic components (Figure 4D). For this analysis, it was assumed that all gel groups behaved elastically, and the compression modulus (E) was calculated from the true stress–strain curve over a strain range of 0–15%, where the R2 value exceeded 0.95 (Figure 4D). Within the tested conditions, one-way ANOVA detected no statistically significant effect in E values across different CaP diameters (*p* = 0.628). The E values for GH gel with CaP sizes of 100 µm, 175 µm, and 250 µm were 13,469 ± 10,668 Pa, 20,260 ± 3493 Pa, and 13,398 ± 6708 Pa, respectively (*n* = 2, mean ± S.D.) (Figure 4E). A clear increasing trend in E was observed among the different gel groups (G < GH < GH-CP), with statistical significance confirmed by one-way ANOVA (*p* = 0.0018). Tukey’s post hoc analysis revealed significant differences between G and GH-CP (*p* = 0.0012). The average mean values of E for G, GH, and GH-CP were 5542 ± 2955 Pa, 9797 ± 2271 Pa, and 15,709 ± 6828 Pa (G (*n* = 8), GH (*n* = 6), GH-CH (*n* = 6), mean ± S.D.), respectively (Figure 4F). Although GH-CP exhibited the highest mean compressive modulus, the difference from GH did not reach statistical significance. These findings indicate that the GH formulation established the principal increase in bulk mechanical competence relative to G, whereas incorporation of CaP maintained this mechanical behavior while producing a consistent numerical trend toward greater resistance to compression.

#### 3.1.4. Expansion Ratio

Expansion ratio after gelation was measured in all three groups (i.e., G, GH, GH-CP) under identical in vitro conditions. Among the three formulations, G showed the smallest degree of expansion, whereas GH consistently exhibited the greatest volumetric increase, and GH-CP in between, indicating improved dimensional stability without eliminating hydration of the composite, throughout the study (Figure 5A). One-way ANOVA revealed significant differences in expansion ratios among the groups (G, GH, and GH-CP) (*p* = 0.0003). Tukey’s post hoc test confirmed statistically significant differences between G and GH (*p* < 0.001), G and GH-CP (*p* < 0.05), and GH and GH-CP (*p* < 0.05). The average expansion ratios were 19.30 ± 11.70% for G, 138.7 ± 47.01% for GH, and 80.03 ± 39.62% for GH-CP (G (*n* = 6), GH (*n* = 5), GH-CH (*n* = 6), mean ± S.D.) (Figure 5B).

#### 3.1.5. Swelling Ratio

Swelling ratio of the GH-CP constructs also remained between that of the G and GH groups throughout the experimental period. Although expansion was lower than that observed in GH, the constructs remained hydrated during culture. Different particle sizes did not show any statistical difference in swelling ratio amongst groups. One-way ANOVA indicated that different CaP diameters did not significantly influence the swelling ratio (*p* = 0.3202). The GH gel with CaP sizes of 100 µm, 175 µm, and 250 µm showed swelling ratios of 44.25 ± 8.17%, 28.63 ± 5.31%, and 39.84 ± 15.03% (*n* = 3, mean ± S.D.), respectively (Figure 5C). A significant difference in swelling ratio was observed among the gel groups (G, GH, and GH-CP) based on one-way ANOVA (*p* = 0.0016). Tukey’s post hoc test revealed significant differences between G and GH (*p* < 0.01) and between GH and GH-CP (*p* < 0.05). The mean swelling ratios were 32.29 ± 11.07%, 53.19 ± 8.153%, and 38.53 ± 10.68% for G, GH, and GH-CP, respectively (*n* = 6, mean ± S.D.) (Figure 5D).

### 3.2. Mechanical Stabilization Supports Cytoskeletal Organization, Cellular Persistence, and Osteogenic Progression

Cell viability in the mechanically stabilized GH constructs was evaluated first, to determine whether needle-orifice delivery and enzymatic cross-linking affected cellular survival. Live/dead staining demonstrated high viability in the G and GH formulations, whereas the H formulation showed a clear reduction in cell survival after in situ gelation (Figure 6A). Viability differed significantly among the three formulations (one-way ANOVA, *p* < 0.0001), with lower viability in H than in G and GH (Tukey post hoc comparisons, both *p* < 0.0001) (Figure 6B). No appreciable loss of viability followed extrusion; so, both the delivery process and gelation chemistry appear cytocompatible.

Qualitatively, the three formulations produced clearly different cell morphologies (Figure 6C). Most cells in G appeared elongated, whereas rounded cells predominated in H. GH did not resemble either group and instead showed an almost even mixture of elongated and rounded cells. The morphometric analysis confirmed these visual differences. Elongated cells comprised 60.3 ± 1.8% of the population in G, compared with 6.7 ± 2.2% in H, while GH contained 49.7 ± 3.8% elongated cells. Rounded cells showed the opposite trend, comprising 39.7 ± 1.8%, 93.2 ± 2.2%, and 50.3 ± 3.8% of the population in G, H, and GH, respectively (*n* = 6; mean ± s.e.m.). The distributions differed among the three formulations (two-way ANOVA, *p* < 0.0001). Additional comparisons indicated that elongated cells were more abundant in G, whereas rounded cells predominated in H (*p* < 0.001).

Robust expression of the osteogenic markers osteocalcin (OCN) and osteopontin (OPN) was observed throughout osteogenic culture (Figure 8C), with fluorescence increasing across G, GH, and GH-CP formulations. OCN consistently exhibited stronger fluorescence intensity than OPN across all hydrogel formulations, although both markers increased following hydrogel combination and CaP incorporation; the quantitative measurements matched these observations. OCN fluorescence intensity increased from 1.570 ± 0.175 in G to 2.516 ± 0.203 in GH and 2.752 ± 0.272 in GH-CP, whereas OPN increased from 0.011 ± 0.0001 in G to 0.348 ± 0.118 in GH and 1.076 ± 0.363 in GH-CP (*n* = 6, mean ± s.e.m.). Two-way ANOVA demonstrated significant effects of hydrogel formulation (*p* = 0.0003) and osteogenic marker (*p* < 0.0001). Post hoc comparisons further demonstrated significantly greater OCN and OPN expression in GH and GH-CP than G (*p* < 0.05), while OCN fluorescence remained significantly greater than OPN within each formulation (Figure 8D). Ki-67-positive nuclei stayed readily detectable throughout early osteogenic differentiation and did not differ significantly among the experimental groups (82.25 ± 20.98, 95.00 ± 28.01, and 104.5 ± 19.02 positive cells for G, GH, and GH-CP, respectively; one-way ANOVA, *p* = 0.42) (Figure 8B).

### 3.3. Mechanical Conditioning Promotes Compartmentalized Matrix Mineralization Beyond Direct CaP Interfaces

Extensive mineral deposition was observed after 28 days of culture by Alizarin Red S (ARS) staining and confocal microscopy. Mineralized regions were distributed along the pre-existing CaP particles and within adjacent hydrogel domains (Figure 9). Many deposits were spatially distinct from the original mineral particles instead of remaining localized to their surfaces (Figure 9A). Higher-magnification imaging further showed mineral accumulation surrounding adherent cells in addition to the incorporated calcium phosphate particles (Figure 9B), suggesting matrix-associated mineral formation beyond the original scaffold interface. The quantitative fluorescence measurements supported these observations. Mean gray fluorescence values under osteogenic culture were 1.044 ± 0.195 for G, 2.136 ± 0.228 for GH, and 4.834 ± 1.367 for GH-CP. The corresponding values under expansion-medium conditions were 1.172 ± 0.202, 1.065 ± 0.165, and 1.294 ± 0.097 (*n* = 6, mean ± s.e.m.). Two-way ANOVA identified significant effects of hydrogel formulation (*p* = 0.0095) and culture condition (*p* = 0.0057). Post hoc analysis demonstrated greater ARS fluorescence in osteogenic GH-CP than in the indicated comparison group G (*p* < 0.01) (Figure 9D).

ARS signals were frequently detected away from the CaP surface, along with directly over the mineral particles, in the spatial intensity maps (Figure 9C); similar findings were also observed relative to the cellular centroids, while mineral-associated fluorescence signals were additionally identified within the hydrogel phase itself. These observations are consistent with secondary mineral nucleation occurring within mechanically stabilized hydrogel microdomains. In other words, the CaP particles appear to establish the initial osteogenic environment, whereas subsequent mineral deposition is not limited to the particle surface alone. Because every mineralization experiment used the same CaP formulation (i.e., 175 μm), these spatial patterns do not reflect particle-size differences and instead point toward a matrix-level mechanical effect.

### 3.4. Ex Vivo Resistance to Compressive Deformation Under Defect-Mimicking Conditions

Upon torquing, CP grafts readily lost their original contour with material displacement beyond the defect margins was also evident, and the graft became progressively less cohesive as loading increased; these changes were consistently observed throughout the defect model (Figure 10E). However, a different response was observed in the GH-CP group. Even under the same loading conditions, the composite largely retained its original shape and remained cohesive within the defect space; little graft redistribution was visible after compression (Figure 10E). Qualitative assessments clearly showed less deformation than in the CP group across the mechanically vulnerable defect configurations (Figure 10E).

According to CBCT quantitative analysis, the structural organization of GH-CP remained intact throughout the study (Figure 10F). Two-factor ANOVA indicated that the deformation ratio (%) significantly varied with different graft materials (CP vs. GH-CP) (*p* = 0.0035) and among different bony defect walls (*p* = 0.0191). Tukey’s post hoc test found significant differences between CP:1-wall defect and CP:3-wall defect (*p* < 0.05), CP:1-wall defect and GH-CP:1-wall defect (*p* < 0.01), as well as CP:1-wall defect and GH-CP:3-wall defect (*p* < 0.01). The mean deformation percentages were 17.72 ± 6.25% for CP:1-wall, 8.12 ± 3.53% for CP:3-wall, 6.32 ± 2.78% for GH-CP:1-wall, and 5.05 ± 2.20% for GH-CP:3-wall (*n* = 4, mean ± S.D.). Under ex vivo compression, the hydrogel-containing constructs retained their overall defect geometry, whereas particulate calcium phosphate alone exhibited greater deformation.

**Figure 11:**
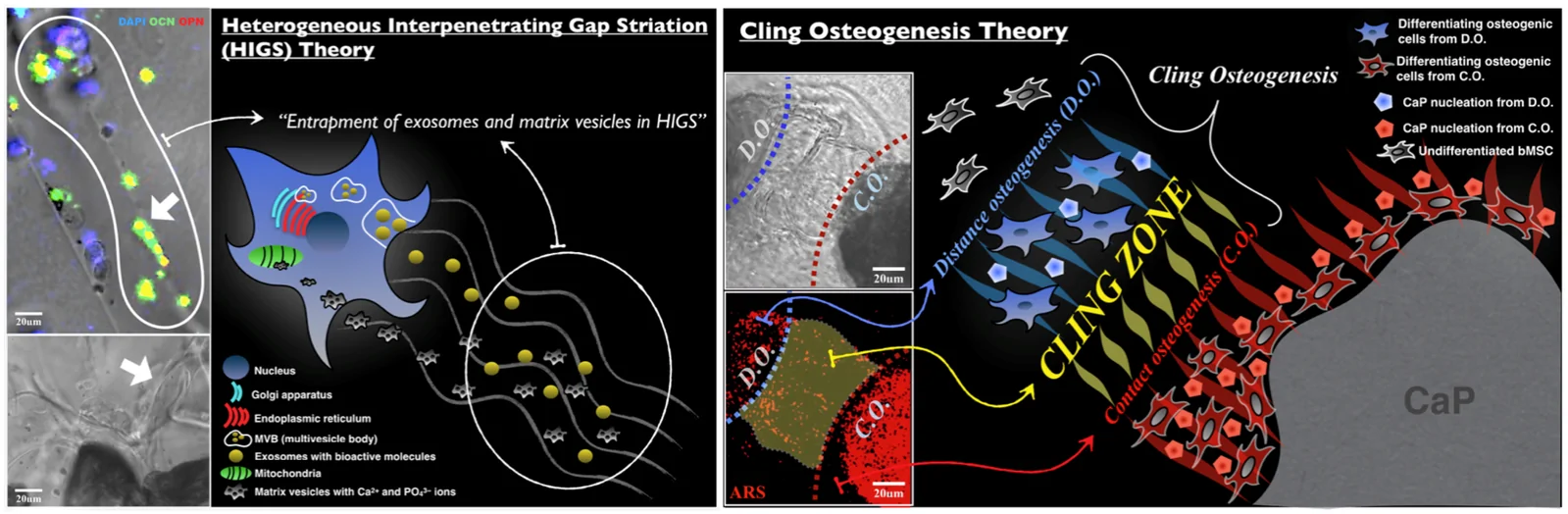
**Integrated mechanobiological concepts of *****heterogeneous interpenetrating gap striation***** (HIGS) and *****cling osteogenesis*****.** HIGS emerges as an intrinsic post-gelation microarchitecture of the cross-linked hydrogel network, characterized by spatially discontinuous, interconnected microdomains independent of mineral incorporation. These matrix-defined organizations seem occasionally associated with vesicle-like nanoscale structures and mineral-adjacent ionic environments, creating spatial conditions supportive of localized osteogenic activity. In parallel, *Cling osteogenesis* describes the preferential adjoined localization and sustained anchorage of osteogenic cells along mineral interfaces, discovered within a mechanically coherent matrix, giving rise to an intermediate *cling zone* that bridges classical contact- and distance-mediated osteogenesis. Together, these theoretical concepts illustrate how hydrogel-mediated containment and early mechanical stabilization organize micro-dimensional space, enabling optical idealization for coordinated cellular anchorage, matrix maturation, and regionally organized de novo osteoid deposition within osteogenic matrices. The conceptual foundations and illustrations were originally developed by Young K. Kim during his doctoral research and documented in his 2019 dissertation [51]; adapted for the present work. White arrow signs represent HIGS.

### 3.5. Heterogeneous Interpenetrating Gap Striation Forms an Intrinsic Post-Gelation Microarchitecture

**Figure 12:**
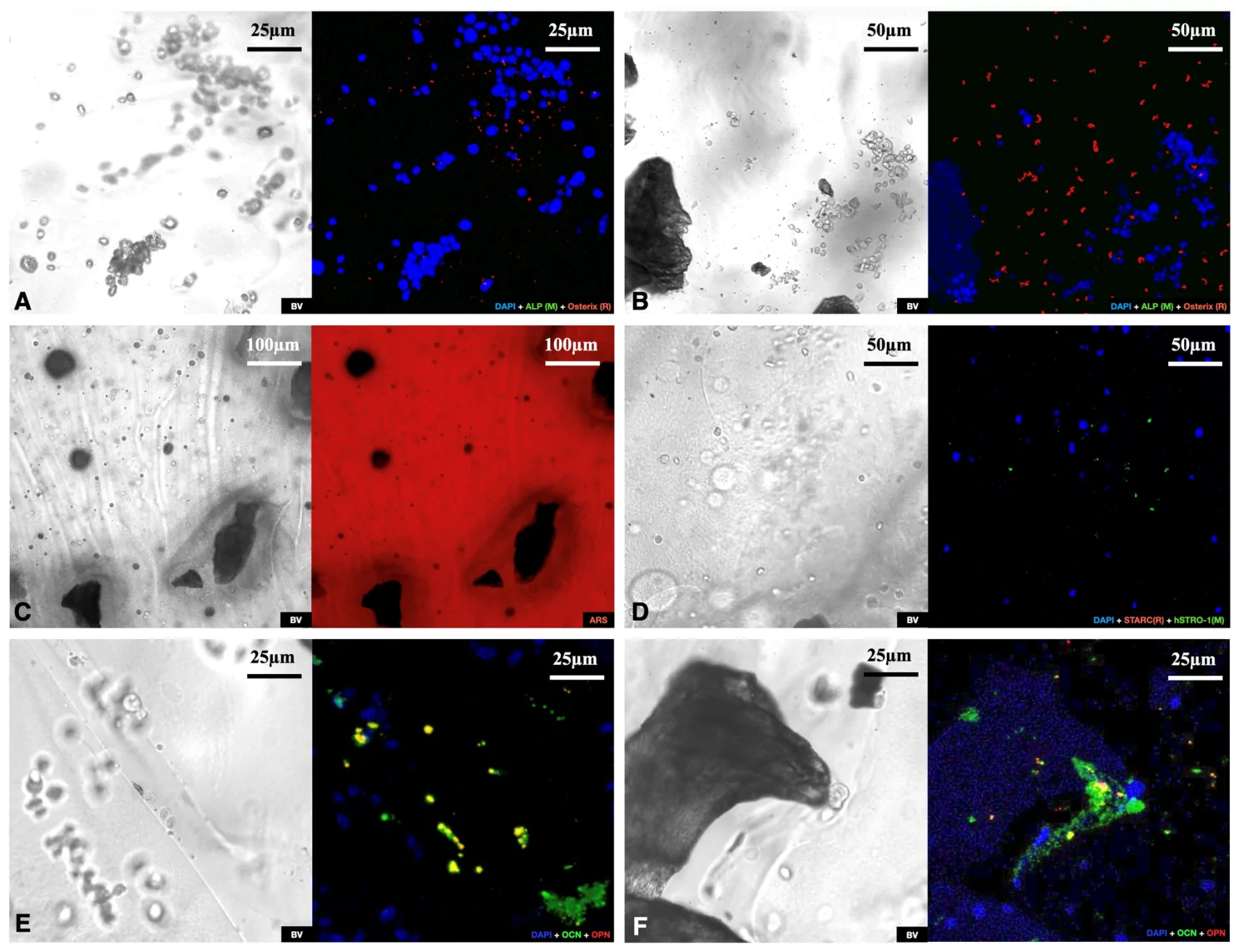
**Spatial concordance of osteogenic, stromal, and mineralization markers within hydrogel substrata reveals a shared pattern organized by *****cling osteogenesis***** and *****heterogeneous interpenetrating gap striation***** (HIGS) microarchitecture.** Confocal immunofluorescence and corresponding bright-field (BF) imaging demonstrate that multiple osteogenesis-relevant readouts exhibit a common non-random spatial distribution within GH–CP constructs. Rather than implying strict temporal staging, this figure emphasizes pattern invariance: marker-positive signals repeatedly localize along mineral-associated *cling zones* and within hydrogel-confined microdomains consistent with HIGS. (**A**,**B**) ALP and Osterix staining highlight osteogenic-associated cellular activity that preferentially concentrates along CaP interfaces and extends into adjacent hydrogel regions consistent with HIGS propagation. (**C**) Alizarin Red S (ARS) reveals localized calcium deposition that follows the same spatial topology, including mineral deposition within hydrogel regions that are not strictly limited to pre-existing CaP surfaces. (**D**) STARC + hSTRO-1 co-staining identifies stromal/progenitor-associated cellular populations and aggregations that are enriched within hydrogel-confined microdomains aligned with HIGS architecture, supporting a structurally permissive niche for cellular clustering and intercellular organization. (**E**,**F**) OCN and OPN staining demonstrate osteogenic matrix maturation signals that remain spatially coupled to *cling zones* and HIGS-associated regions, reinforcing that osteogenic lineage progression and matrix deposition are organized by the same interstitial topology. Collectively, these panels provide immunohistological support for the central model proposed in Figure 11: osteogenic activity within rigidity-preserved hydrogel–mineral systems is spatially orchestrated by (i) mineral-interface anchorage (*cling osteogenesis/cling zones*) and (ii) intrinsic hydrogel microarchitecture (HIGS), enabling coordinated cellular aggregation and osteogenic matrix deposition across both mineral-adjacent and hydrogel-contained compartments.

A recurring structural pattern became apparent during microscopic examination of the post-gelated hydrogels; throughout the polymer network, discontinuous yet interconnected gap-like striations were repeatedly observed (bright-field views in Figure 7, Figure 8, Figure 9 and Figure 12). We refer to this micro-architectural phenomenon as heterogeneous interpenetrating gap striation (HIGS) (Figure 11). The same pattern appeared in both gel-only and hydrogel–mineral formulations, indicating that it develops within the enzymatically cross-linked hydrogel itself, not solely from mineral incorporation. Cell clusters and newly deposited matrix were frequently located within these interconnected microdomains.

At higher magnification, the gap-like striations remained continuous rather than terminating as isolated spaces (bright-field views in Figure 7C and Figure 8A,C); the surrounding matrix was partitioned into smaller compartments while the overall continuity of the hydrogel was retained. Similar microarchitectural patterns were observed in independent specimens (Figure 12). Small vesicle-like nanostructures (see Section 4.3) were also present adjacent to mineral-associated cellular interfaces in several regions (Figure 11 and Figure 12E). Altering the needle-orifice diameter or delivery geometry produced no obvious change in the overall spatial organization.

The temporal microscopic observations was also validated for its sequential events (Figure 7). The HIGS emergence (Figure 7C) occurred concomitantly with cell migration within the hydrogel and toward mineral surfaces (Figure 7D–F), followed by extension into intersubstratal regions where osteoid and calcified matrix subsequently developed (Figure 7G–I). Collaterally, the microdomains remained visible within the surrounding hydrogel. Viewed together, osteogenesis appears to progress within a structurally heterogeneous hydrogel microarchitecture, not being confined to the immediate mineral interface. Hence, the GH-CP construct seemed to proceed a structural integration, along with a progressive heterogeneous microarchitectural morphogenesis. This phenotype does not reflect random pore formation, but rather a sophisticatedly organized hydrogel–mineral topography that likely contributes to nutrient transport, cellular infiltration, and subsequent osteogenic activity (see Section 4.2 and Section 4.3).

### 3.6. Cling Osteogenesis Defines a Mineral-Associated Intermediate Osteogenic Zone

Mineral-depositing cells were frequently observed along the surfaces of the particles, with continuous stratifying extensions, intertwined with HIGS regions (see Section 3.5), into the surrounding hydrogel (Figure 7D–F). Within the same microscopic fields, osteogenic marker expression appeared to be greatly scattered within the matrix regions; in other words, de novo bone formation was likewise not limited to the immediate particle surface but frequently extended into the adjacent hydrogel (Figure 8C). The cells remained attached to the mineral surface, yet the mineralized matrix often appeared sporadically, interjecting into the surrounding distant osteoid through the HIGS channels from the original CaP topographic interface. Because this spatial pattern turned up repeatedly (Figure 9), we propose the concept of *cling osteogenesis* (Figure 11), where intricate structural arrangements of mineral-associated cells, osteoids, and calcified matrix deposits coexist within interconnected hydrogel compartments as a junctional zone of early osteogenic regions between contact and distant osteogenesis (see Section 4.4).

### 3.7. Spatial Concordance of Osteogenic and Stromal Markers Reinforces HIGS- and Cling Zone-Guided Organization

To examine whether this spatial organization reflects a recurring biological pattern, as opposed to isolated morphological observations, supplemental immunohistological analyses were performed across multiple osteogenesis-relevant markers. Across markers representing osteogenic activity (ALP, Osterix), matrix mineralization (ARS), stromal/progenitor-associated identity (STARC, hSTRO-1), and matrix maturation (OCN, OPN), signal localization exhibited a strikingly consistent spatial distribution of the geometric structural phenomena above (Figure 12). Marker-positive regions and mineral deposits were repeatedly observed along mineral-associated *cling zones* (Figure 11) and within hydrogel microdomains corresponding to the HIGS architecture; a similar spatial arrangement was observed regardless of whether the markers reflected osteogenic differentiation, matrix maturation, stromal identity, or mineral deposition. Rather than appearing as isolated artifacts, the same distribution pattern held across every specimen examined. HIGS-associated microdomains (see Section 3.5), together with mineral-associated extra-cellular anchorage (i.e., *Cling osteognesis*), seemed to set a common dimensional acquisition in which cellular aggregation, lineage progression, and localized matrix deposition occur within this distinctive zone of hydrogel substratum (see Section 4.4).

## 4. Discussion

### 4.1. Injectable Dual-Syringe Delivery Through a Needle-Orifice, Early Structural Stabilization, and Mechanotransducive Microenvironment

The present findings indicate that the early organization of osteogenic tissues within injectable hydrogel–mineral systems depends heavily on the microarchitecture established soon after material placement (Figure 7). During the earliest stages of healing, periosteal recoil, soft-tissue closure, and local muscular activity expose the graft to compressive and shear forces while cells begin to attach, spread, and commit to an osteogenic lineage [1,2,3,5]. Under these conditions, an injectable material may maintain good cell viability yet still provide an environment that changes too readily to support organized osteogenic development and matrix formation if mechanical stability is not established early [4,6].

In our system, rapid stiffening followed needle-orifice delivery (Figure 3) and was accompanied by preservation of the local structural organization during the early post-delivery period (Figure 2 and Figure 6). Cell–matrix continuity remained intact as the construct stabilized (Figure 7), coinciding with the stage at which mechanotransductive signaling becomes increasingly important [6,37]. These observations suggest that the period immediately after injection helps establish the environment in which subsequent tissue regeneration occurs.

The mechanical measurements first showed that storage modulus increased significantly from G to both GH and GH-CP, while GH-CP showed a further numerical increase over GH that did not reach statistical significance. Compression testing similarly showed an upward trend in modulus across the same groups (Figure 3 and Figure 4). In contrast, the swelling and expansion experiments demonstrated that CaP incorporation reversed the hydrogel’s post-gelation volumetric behavior (Figure 5), while GH exhibited the greatest expansion, consistent with the highly hydrated and mechanically compliant character of the combined gelatin–hyaluronan network [29,30]. Critically, addition of CaP reduced this expansion without returning the construct to the substantially lower level observed in G, producing the sequential relationship of swelling/expansion ratio as G < GH-CP < GH. Collectively, these results point to a *mineral-mediated mechanical buffering effect*, in which CaP partially counteracted the volumetric expansion introduced by the GH formulation while contributing to early viscoelastic and dimensional stabilization of the construct. The sequential comparison among G, GH, and GH-CP justifies that the final volumetric behavior reflects a balance between hydrogel-driven expansion and mineral-mediated structural restraint.

A notable discovery of the present study, therefore, was the opposing influence of the hydrogel formulation and CaP incorporation on post-gelation construct behavior. To our knowledge, this sequential behavior (G → GH → GH-CP) has not been specifically analyzed. Previous studies have generally reported reduced swelling after introducing calcium phosphate into hydrogels and have attributed this to stronger hydrogel–mineral interactions, restricted polymer mobility, increased cross-linking density, or partial occupation of the hydrogel pore network [52,53]. Our findings are consistent with those mechanisms but additionally suggest that the mineral phase (GH-CP) may counteract the expansion introduced by the composite hydrogel formulation itself (G → GH). One possible explanation is that incorporation of CaP established localized particle–polymer interfacial domains that mechanically stabilized the surrounding hydrogel network while restricting its expansion. Hydroxyapatite particles are known to adsorb macromolecular chains and can serve as non-covalent bridging interfaces in soft materials [54], making this interpretation plausible. Although these interactions were not directly examined in the present study, they provide a reasonable mechanistic explanation for the reduced volumetric expansion (Figure 5B,D) despite mechanically stabilized behavior observed in the GH-CP group (Figure 3F and Figure 4F).

Importantly, the swelling behavior paralleled the volumetric expansion findings, providing an independent verification of the same structural response. Whereas GH exhibited the greatest swelling, GH-CP maintained an intermediate swelling profile (i.e., not statistically different from G), indicating that CaP moderated hydrogel expansion without eliminating its intrinsic hydration capacity. This concordance between the expansion and swelling analyses strengthens the interpretation that the mineral phase restrained excessive dimensional change while preserving a hydrated construct [17,19]. Consistent with these physical observations, cellular viability, osteogenic marker expression, and matrix mineralization remained evident throughout the construct (Figure 8, Figure 9 and Figure 12), indicating that the moderated swelling response did not compromise the biological activity required for osteogenesis. The same volumetric behavior was recapitulated under ex vivo defect conditions (Figure 10), in which GH-CP preserved the graft geometry against external compression. Together, reduced post-gelation expansion, maintenance of the higher mechanical values observed in the composite, and preservation of geometry under ex vivo loading supported the dimensional stability of the composite during the early period after placement [14,15,16]. These observations suggest a potential clinical advantage during GBR, where preservation of graft geometry together with early mechanical stability may improve space maintenance within the subperiosteal pouch under physiologic dimensional changes (see Section 4.5).

The present hydrogel–mineral construct differs from many injectable osteogenic biomaterials in a way that became more apparent as the study progressed. Injectable hydrogel–CaP systems must satisfy practical delivery requirements in addition to providing adequate post-gelation mechanical support. Recent reviews have emphasized that injectability depends on the interplay among viscosity, particle loading, injection force, needle geometry, gelation kinetics, and phase stability rather than on any single material property [55,56]. Likewise, successful composite formulations require balancing the handling characteristics of the hydrogel carrier with the biological contribution of the mineral phase instead of simply focusing on material incorporation [55]. These considerations guided the formulation strategy adopted in the present study. Most injectable hydrogels provide a hydrated environment that supports cell survival and molecular transport, whereas CaP-based materials contribute mineral interfacial support and modify the mechanical behavior of the composite [18,20,29]. In the present system, the higher mean rheological and compressive values observed after CaP incorporation also justify that such a mechanical contribution remains possible, although the differences relative to GH were not statistically significant.

Additionally, these functions are often combined by incorporating bioactive molecules or growth factors [27,33,57]. In our study, such micro-preservation of construct architecture was closely associated with the biochemical environment established within the injectable hydrogel itself without any extrinsic bioactive enhancers, except the osteogenic medium. Although the material remained injectable, rapid stabilization after gelation preserved the overall structure (Figure 2A) while permitting continued cell survival, spreading, and reorganization within the matrix (Figure 6, Figure 7, Figure 8 and Figure 9).

The pilot sedimentation study initially demonstrated that CaP particles remained dispersed within the HA–Tyr precursor throughout the working period before injection, thereby minimizing particle settling prior to mixing (Figures S1 and S3). Although filter pressing during extrusion was beyond the scope of the present study, no obvious phase separation or particle retention was observed during routine specimen preparation (Figure 2 and Figure S2). This combination was repeatedly associated with the subsequent appearance of localized osteogenic microdomains and matrix mineralization (Figure 8). Although the present work does not establish a causal relationship, it raises the possibility that maintaining the initial physical microenvironment immediately after delivery with enhanced microstructures may influence forthcoming osteogenic lineages to a greater extent than has generally been appreciated [1,2,37].

Morphological observations were obtained throughout the study using gross examination, optical microscopy, confocal microscopy, and ex vivo CBCT imaging rather than through a single dedicated experiment. Hence, these evaluations consistently demonstrated uniform CaP distribution after injection, preservation of hydrogel continuity, and close association between the hydrogel and mineral phases. Although the present study emphasized structural morphology associated with injectable delivery and regenerative performance, more detailed molecular and physicochemical characterization of the hydrogel–CaP interface is currently in progress and will be reported in future studies.

Beyond its potential contribution to the overall mechanical behavior of the composite, the mineral phase provided interfacial surfaces along which cells repeatedly attached and organized (Figure 7D–I), consistent with previous observations that mineral interfaces influence cytoskeletal organization and osteogenic differentiation [18,19]. Throughout the present study, osteogenic cells were repeatedly observed along these interfaces, maintaining organized cytoskeletal morphology (Figure 8A) and expressing osteogenic markers (Figure 8C) consistent with sustained mechanotransductive activity [2,4]. Viewed together, these observations suggest that early physical organization and stabilization of the construct preceded the later biochemical events associated with tissue maturation.

The spatial relationship observed between mechanical organization and mineral-associated cellular behavior also deserves consideration [58]. Although mineral-derived ionic signaling was not measured directly, mineral surfaces, cellular organization, and localized matrix deposition repeatedly appeared within the same regions of the construct (Figure 9). This recurring spatial association suggests that preservation of the construct architecture may help maintain localized mineral-associated interactions within the surrounding hydrogel in which the mineral phase may function as more than a passive osteoconductive substrate (see Section 4.4) by participating in the formation of a spatially organized mechanotransductive microenvironment [17,18].

### 4.2. HIGS as a Mechanically Preserved Intrinsic Osteogenic Microarchitecture

One feature that repeatedly emerged in the present study was the presence of *heterogeneous interpenetrating gap striation* (HIGS) (Figure 11) in all gel casts, with its frequent presence order of G < GH < GH-CP (bright-field views on Figure 7, Figure 8, Figure 9 and Figure 12). Because this microarchitecture was identified regardless of whether CaP particles were incorporated, it appears to arise from the mechanically stabilized hydrogel network itself, rather than exclusively from the osteoconductive CaP particles. The conceptual foundations underlying HIGS and the subsequently described *cling osteogenesis* (see 4.4.) were originally developed during Young K. Kim’s doctoral research and documented in his 2019 dissertation [51].

The original experimental configuration focusing primarily on handling characteristics and injectability through needle-orifice module (see Section 4.1), as characterized with 10 mg/mL of CaP, turned out providing sufficient intersubstratum optical status to permit direct microscopic observation of these structural interactions, which might have been obscured at higher particulate loading or with more densely packed mineral phases in conventional investigations. Because the CaP particles occupied dispersed fraction of the construct in our study, sufficient interstitial hydrogel space remained to visualize cellular and matrix organization within the substratum, actualizing the discovery of HIGS and *cling osteogenesis* (see Section 4.4) beyond the immediate hydrogel–mineral interface.

Following CaP incorporations, HIGS appeared more distinct following CaP incorporation (Figure 8 and Figure 12). Since GH-CP exhibited less volumetric expansion than GH (Section 4.1), this raises the possibility that reduced expansion and swelling generated greater local tensocompression within the intersubstratum, thereby promoting more pronounced or invaginated striations during hydration. The higher mean rheological and compressive values observed in GH-CP may also have contributed to preservation of this local architecture, though this mechanical gap over GH was not statistically confirmed.

Microscopic examination further revealed that HIGS did not represent homogeneous polymer network (Figure 7). Instead, the hydrogel contained discontinuous yet interconnected gap regions with variable interstitial spacing. Such structural heterogeneity could influence the movement and local retention of soluble factors, as well as the organization of cells within the surrounding matrix [36,59,60,61]. At present, the molecular basis of HIGS remains unresolved, and the present observations should therefore be viewed primarily as a reproducible structural phenomenon rather than a fully defined biological mechanism [5,12].

One interesting observation was the appearance of localized cellular aggregates in a subset of the hydrogel constructs after culturing (Figure 8 and Figure 12), preceding more symmetric cellular distribution initially observed after the immediate gelation (see Section 4.1). These aggregates were more frequently identified within G (Figure 8A) or immediately adjacent to HIGS-associated microdomains (Figure 12E), although this spatial relationship was evaluated qualitatively rather than quantitatively. Few of these aggregates also appeared in GH constructs without CaP particles or in CaP-free regions within the GH-CP constructs (Figure 8C), while cells immediately adjacent to CaP particles generally remained more evenly dispersed. This pattern was also compatible with the mechanical characterization, in which both GH and GH-CP exhibited significantly greater storage modulus than G, while GH-CP demonstrated higher mean storage and compressive moduli than GH. It is therefore possible that cells located within the more mechanically compliant, CaP-free hydrogel regions, which may also have permitted local cellular rearrangement, were able to reorganize gradually during culture, whereas mineral-associated regions may have provided greater local structural restraint. Similar relationships between hydrogel mechanics and cell organization have been described previously [31,37,62].

Cellular migration through a plasticized, tangible hydrogel substratum could likewise have generated these trace-like remnant microstructures, representing another possible etiologic explanation for HIGS formation. The presence of aggregates within CaP-free regions of GH-CP constructs additionally suggests that the surrounding hydrogel remained sufficiently permeable for limited cellular movement after gelation. Interestingly, when these localized cellular condensations were observed together with visually identifiable HIGS regions, the spatial distribution of osteogenic marker expression (Figure 12E,F) and mineralized deposits (Figure 9A and Figure 12C) strikingly appeared to follow the same geometric pattern within its entity. Although this relationship was not quantified, the recurring spatial alignment among HIGS microarchitecture, cellular organization, osteogenic marker, and mineralized deposit localization formed one of the principal observations that motivated the HIGS concept. Whether these aggregates resulted from migration, localized proliferation, matrix contraction, or a combination of these events cannot be determined from the present study. For that reason, regions containing obvious cellular condensations were not included in the quantitative image analysis, which instead used representative fields with relatively homogeneous-distributed sites (Figure 9B). Future work using longitudinal live-cell imaging together with spatial mapping of local mechanical properties will be necessary to determine whether these geometric relationships reflect an active mechanobiological process or simply a recurring structural association.

Although the biological significance of HIGS remains unclear, interconnected microdomains, attached cellular clusters, localized matrix deposition, and adjacent mineral interfaces were found together in each specimen we examined. Accordingly, these observations suggest that HIGS represents more than a microscopic curiosity; it may provide a new in vitro experimental armamentarium that enables comprehensive investigation of detailed cellular aggregation dynamics, localized retention of mineral-associated signals, and matrix deposition mechanisms that might otherwise be veiled in conventional experiments.

### 4.3. Vesicle-like Structures Within HIGS Microdomains and Localized Intercellular Signaling

The repeated association between HIGS regions and extracellular vesicle-like structures (Figure 8C, Figure 11 and Figure 12E) suggests that these microdomains may provide localized environments favorable for intercellular communication during early osteogenic organization. Although definitive identification of these structures was beyond the scope of the present study, their morphology and consistent spatial distribution suggest that biologic interactions may preferentially occur within these HIGS-associated regions among mineral-associated cells.

Occasional nanoscale vesicle-like structures were noted in higher-magnification images of confined hydrogel–mineral interfaces (Figure 12F). These structures were most often encountered in areas where cellular aggregation and early matrix deposition were already present (Figure 12C,E). These observations may be biologically relevant because the HIGS architecture appears to maintain structural continuity among neighboring cells, mineral surfaces, and newly deposited extracellular matrix (Figure 7). Should this spatial organization fail to persist during the early post-gelation period, the boundaries between these localized regions would likely become less apparent, and the corresponding intercellular relationships might no longer be as readily recognized by microscopy. This possibility is consistent with previous reports indicating that extracellular vesicle trafficking and mechanosensitive communication depend strongly on local matrix organization, spatial confinement, and biomechanical stability [63,64].

Growing evidence from skeletal regeneration studies has linked extracellular vesicles and exosome-associated signaling with osteogenic differentiation, matrix mineralization, angiogenic recruitment, and mechanotransductive communication [65,66,67]. Mesenchymal stem cell-derived extracellular vesicles transport osteoregulatory microRNAs, signaling proteins, and mineralization-associated mediators that influence neighboring cellular behavior and extracellular matrix maturation during bone formation [66,67]. In the present study, HIGS-associated microdomains may generate confined hydrogel microenvironments that favor local retention of vesicle-associated signaling during the early stages of osteogenesis (Figure 11). In the present constructs, hydrogel and mineral phases remained closely apposed after stabilization (see Section 4.1); this arrangement may allow neighboring cells to remain in close proximity while reducing local diffusional dispersion, a setting that could favor localized signaling as early matrix organization develops [1,2].

During microscopic examination, vesicle-like structures were repeatedly observed adjacent to mineral-associated cellular interfaces (Figure 9C and Figure 12F) as well. The repeated spatial association between these structures and CaP particles suggests that the mineral phase may coincide with localized regions of biologic activity in addition to serving as an osteoconductive attachment surface (see Section 4.4). The present observations do not distinguish between localized accumulation of signaling molecules and preferential cellular organization around mineral interfaces. Nevertheless, coordinated osteogenic activity was consistently observed throughout the GH-CP specimens, including matrix deposition extending beyond regions of direct particle contact (Figure 9A). Comparable spatial relationships between CaP-rich environments and osteogenic signaling have likewise been described in previous osteoconductive systems, where local mineral organization influences cell adhesion, matrix maturation, and signaling activity [18,68].

Because these observations originated from descriptive microscopic evaluation rather than experiments specifically designed to investigate extracellular vesicles, additional studies incorporating transmission electron microscopy, CD63/CD81 immunolabeling, nanoparticle tracking analysis, and spatial transcriptomic approaches will be necessary to determine whether HIGS-associated microdomains preferentially retain extracellular vesicles or support localized signaling exchange. Current extracellular vesicle guidelines also require molecular and ultrastructural characterization before vesicle-like structures can be conclusively classified as exosomes or extracellular vesicles [64]. Taken together, these observations suggest that preservation of hydrogel microarchitecture may help maintain spatially organized patterns of intercellular communication within mechanically stabilized osteogenic compartments, although direct experimental confirmation of this mechanism remains a critical objective for future work.

### 4.4. Cling Osteogenesis and Spatially Constrained Osteogenic Patterning

Microscopic examination repeatedly identified mineral-depositing osteogenic cells at both hydrogel intaglios and embedded CaP interfaces within the mechanically stabilized constructs (Figure 7). In many fields of view, these cells remained attached to mineral particles while simultaneously extending into the surrounding hydrogel. Their distribution was not limited to the immediate mineral surface, nor was it restricted to more distant tissue-equivalent regions (Figure 9A). This spatial arrangement differed from the classical descriptions of contact osteogenesis [68], in which bone forms directly on a surface, and distance osteogenesis, where bone formation progresses from adjacent host tissues. Across the present specimens, this recurring organization was consistently observed under conditions that combined distributed mineral interfaces with a mechanically stabilized matrix. Because this geometric configuration was repeatedly observed, we use the term *cling osteogenesis* as a descriptive label for the observed positioning pattern (Figure 11).

Such microarchitectural pattern was the easiest to recognize in constructs in which mineral particles were distributed throughout the hydrogel rather than limited to a single interface. In these specimens, cells frequently maintained contact with both the surrounding matrix and neighboring mineral particles while preserving organized cytoskeletal morphology (Figure 7). Markers of osteogenic differentiation were commonly detected in regions located between larger mineral surfaces and adjacent hydrogel compartments, rather than appearing exclusively at one location or the other (Figure 8C). Comparable spatial organization was difficult to appreciate in constructs lacking either cohesive hydrogel support or distributed mineral interfaces. For this reason, *cling osteogenesis* is presented here as a descriptive structural observation based on reproducible positioning configurations, not as a separate biological mechanism.

Shortly after placement, the construct resisted early deformation while maintaining HIGS together with the overall spatial arrangement of mineral surfaces and surrounding cells. The architecture showed little visible alteration during the initial post-gelation period (week 0–1), followed by progressive, simultaneous HIGS morphogenesis and cellular migration (week 1–2). Mineral accumulation likewise appeared preferentially around cell-associated regions, consistent with localized nucleation and subsequent maturation within these mechanically stabilized compartments or in proximity of CaP particles (week 2–4) (Figure 7). Such temporal progression of osteogenic differentiation formed the basis for the concept of *cling osteogenesis*: if mineral deposition occurred in different regions simultaneously and ultimately united, intervening joining regions would inevitably be expected.

The present system unexpectedly enabled visualization of geometric osteogenic phenomena that are rarely accessible in conventional experimental or clinical settings. Neither HIGS nor *cling osteogenesis* (Figure 11) was an anticipated finding at the outset of this investigation. Both became recognizable only because the mechanically stabilized, optically permissive hydrogel preserved the spatial relationships among cells, mineral particles, and newly deposited matrix over time. Rather than resulting from experiments specifically designed to interrogate these phenomena, the observations developed during routine microscopic evaluation of the constructs themselves. Their recognition may therefore reflect the unusual accessibility of these spatial realms, as opposed to becoming obscured by tissue collapse, rapid remodeling, or surgical variability in vivo.

Similar spatial relationships were observed throughout the supplemental immunohistochemical analyses (Figure 12). Osteogenic activity, stromal/progenitor-associated populations, and mineral deposition repeatedly localized to interstitial regions corresponding to *cling zones* and HIGS-associated microdomains. This distribution was observed across the different markers examined, indicating that this unique microarchitecture was maintained throughout the analyses. The present study was not designed to define the molecular mechanisms responsible for these observations. Instead, it provides a structural foundation for future investigations into extracellular vesicle signaling, ion transport, mechanotransductive pathways, and other processes governing early osteogenic organization.

Consequently, *cling osteogenesis*—which may likewise already exist in our physiological and anatomical systems—empirically depicts the recurring spatial dimensions among mineral-associated cells, adjacent hydrogel regions, and the developing osteogenic matrix, as a structural coupling mechanism between adjacent osteogenic compartments.

### 4.5. Clinical Implications of GH-CP

Although the present study was designed to investigate the structural and biological behavior of the GH–CP construct, several observations clearly justify practical implications for injectable bone grafting. Critically, immediate volumetric preservation of the early post-placement architecture greatly potentiates its advantage of graft performance before vascularization and tissue integration cascades.

In a surgical perspective, the earliest period after graft placement may be more essential than it first appears. Before vascular/fibrin ingrowth and cellular integration reinforce the graft, particulate materials in poorly contained defects can lose their original contour under periosteal closure and surrounding soft-tissue forces. By comparison, the hydrogel–mineral construct showed a different pattern; it became mechanically cohesive soon after placement, which was also evident in the ex vivo experiments, yet remained permissive for ongoing cellular activity. Across the tested conditions, the construct generally retained its over-under geometry better than particulate-only grafts, which instead underwent comparable collapse (Figure 10). Clinically, such advantage may become most noticeable in defects offering limited structural support, where early deformation would be expected to occur before substantial biologic integration. Under these circumstances, the immediate post-delivery cohesion observed in GH-CP brings a practical advantage by preserving graft geometry during the interval before biologic integration [7] because materials that remain mechanically coherent while supporting cellular function may therefore provide conspicuous benefits in anatomically restricted defects [32,33,57]. This physical characteristic introduces an alternative surgical paradigm. Rather than considering periosteal tension a liability after injection, the unified construct may derive further enhanced stabilization through its immediately plasticized internal micro-architecture, within the surrounding anatomical tension paradoxically reinforcing its macro-structural rigidity.

Subsequently, such interventions may be most applicable to procedures performed within confined anatomical spaces, where an injectable graft must adapt to a predefined geometry immediately after delivery. Examples include flapless periodontal and peri-implant regeneration, minimally invasive tumor enucleation surgery, craniofacial and maxillofacial reconstruction, orthopedic bone defects, spinal fusion procedures, and selected micro-reconstructive applications in plastic surgery. Despite the differences among these procedures, they share one very common denominator. The graft is introduced into a space that already imposes mechanical constraints through surrounding soft tissues, periosteal tension, or the topography of the defect itself, and deformation under these early constraints may alter the original distribution of mineral particles, the surrounding hydrogel network, and the spatial relationship established among the material components at the time of placement. In the present system, the hydrogel and CaP particulates appeared to preserve this organization while remaining permissive for continued cellular migration, differentiation, and mineralization. Although the present study does not demonstrate in vivo clinical efficacy, it raises the possibility that maintaining the initial three-dimensional architecture may itself denote a useful design objective for minimally invasive injectable regenerative therapies delivered through a needle orifice.

### 4.6. Experimental Limitations and Future Mechanobiological Investigations

#### 4.6.1. Study Limitations

The present findings establish a reproducible structural and mechanical geometry linking early microarchitectural stabilization to organized osteogenic behavior across independent analyses. This investigation was not, however, designed to establish direct causal relationships among early microarchitectural stabilization, HIGS formation, *cling osteogenesis*, and subsequent osteogenic behavior. These observations should therefore be interpreted as a basis for micro-mechanobiological hypotheses rather than as confirmation of a single underlying mechanism. In addition, the recurrence of HIGS and *cling osteogenesis* across specimens was assessed through representative image review rather than blinded or quantitatively scored analysis (e.g., systematic incidence counts across fields or specimens); the reported consistency therefore reflects qualitative pattern recognition and should be interpreted accordingly until a standardized scoring approach is applied in future work.

Mechanotransductive pathways responsible for the spatial osteogenic organization observed in the present hydrogel–mineral system were not examined directly in a molecular-quantifying-mechanistic level. As a result, the individual roles of integrin-mediated adhesion, cytoskeletal tension transfer, focal adhesion maturation, and downstream mechanosensitive transcriptional regulators remain unresolved. Clarifying how these processes interact within mechanically stabilized hydrogel–mineral constructs will require dedicated mechanistic studies in the future. As noted in Section 2.15, image-based quantification (OCN/OPN, ARS, Ki-67, and morphometric analyses) was not blinded to group identity. Acquisition settings were held constant within each staining experiment (Section 2.12), but unblinded analysis cannot fully exclude observer-related bias—a factor to weigh alongside the magnitude of the reported group differences.

Extracellular vesicle-like structures were also identified within HIGS-associated microdomains, yet their molecular identity and biologic function remain unresolved. The present observations were based on morphology alone and therefore cannot distinguish extracellular vesicles from other nanoscale extracellular structures. Future investigations combining transmission electron microscopy, nanoparticle tracking analysis, CD63/CD81 immunolabeling, proteomic profiling, and spatial transcriptomics will be necessary to determine whether mechanically preserved HIGS compartments preferentially retain extracellular vesicles or facilitate localized intercellular signaling during osteogenesis [64].

Although HIGS consistently appeared adjacent to CaP-rich regions, the contribution of mineral-derived ionic activity to its formation remains uncertain because the local ionic environment was not measured directly. Future quantitative ion mapping and high-resolution spatial imaging may help distinguish whether HIGS arises primarily from structural stabilization of the hydrogel or from a coupled mechanochemical interaction involving matrix mechanics, mineral-associated ionic activity, and osteogenic organization.

The ex vivo deformation model provided controlled insight into the mechanical behavior of the graft immediately after placement, although it could not fully recapitulate the biologic complexity of wound healing dynamics in vivo. Long-term clinical studies will therefore be required to determine how the observed structural organization evolves during neovascularization, matrix remodeling, osteogenic maturation, and host tissue integration.

#### 4.6.2. Future Mechanotransducive Investigations

The current findings establish a useful structural foundation for investigating spatially organized osteogenesis under mechanically controlled regenerative conditions. Further work should now determine whether the relationships observed here persist across different hydrogel chemistries, mineral compositions, mechanical environments, and in vivo stages of bone regeneration.

Looking across the observations presented in this study, one recurring pattern became apparent. Preservation of structural organization immediately after hydrogel delivery consistently coincided with organized osteogenic behavior during later stages of regeneration. HIGS-associated microdomains, *cling osteogenesis*, and localized patterns of intercellular signaling were illustrated repeatedly, despite being evaluated using different experimental approaches. Whether these findings represent separate biological events or different expressions of the same mechanotransducive process cannot yet be determined. Their repeated occurrence, however, suggests that these observations are unlikely to be independent and may instead coalesce into a common geometric intersection of micro-mechanistic-osteogenic principles.

## 5. Conclusions

The present study developed a fully injectable hydrogel–mineral construct that maintained homogeneous delivery of calcium phosphate particles and cells through a needle-orifice system while rapidly establishing a cohesive hydrogel network after extrusion. Incorporation of CaP particulates preserved the internal structural rigidity of its architecture, reduced volumetric expansion relative to the gel alone, and maintained mechanical competence during the early post-gelation period. Within this stabilized microenvironment, distinct cellular organization, matrix mineralization, and HIGS were consistently observed across independent experiments. Although these findings do not establish a causal mechanism, they suggest that preservation of the initial physical microarchitecture may influence subsequent osteogenic differentiation. The recurring unique geometric pattern among mineral-associated cells and progressively orchestrating de novo calcium matrix is described here as *cling osteogenesis*, a descriptive term for the adjoining dimensional zones documented in the present model. Collectively, these observations identify early post-delivery microstructural preservation as a design consideration that may merit further attention in the development of injectable bone graft systems for more sophisticated experimental investigation and surgical application.

## Figures and Tables

**Table 2:** Calcium phosphate particle sizes used for each experimental application, with a summary of the CaP particle sizes employed throughout the study. Three particle sizes (~100, ~175, and ~250 μm) were initially evaluated during injectability and particle-distribution optimization. Based on these preliminary studies, approximately 175 μm CaP particles were selected for all subsequent biological and ex vivo experiments.

Experiment	CaP Particle Size
Injectability optimization	~100, ~175, and ~250 μm
Particle distribution optimization	~100, ~175, and ~250 μm
Rheological characterization	~100, ~175, and ~250 μm
Compression testing	~100, ~175, and ~250 μm
Swelling analysis	~100, ~175, and ~250 μm
Biological assays	~175 μm
Ex vivo deformation analysis	~175 μm

## Data Availability

The datasets generated and/or analyzed during the current study are available from the corresponding author on reasonable request.

## Supplementary Materials

The following supporting information can be downloaded at: https://www.mdpi.com/article/10.3390/jfb17080389/s1. Figure S1. Sedimentation phenomenon (**A**) Pre-gelation CaP sinking phenomenon; (**B**) Quantitative analysis of clogging issue (C = vertical distance of sinking CaPs, G = total vertical distance of gel); (**C**) Inclusion pie graphs. Figure S2. (**A**) Schematic drawing of the dual syringe model; (**B**) First prototype; (**C**) Second prototype; (**D**) Final syringe system; (**E**) Too rigidly cross-linked GH gel; (**F**) Too weakly cross-linked GH gel; (**G**) Cross-linked GH-CP; (**H**) Fine tuning [HRP] and [H_2_O_2_]. Figure S3. (**A**) Sequential experimental photographs of pre-gelation CaP sedimentation test; (**B**) Complete sinking seconds (*n* = 7).
