# Supplementary material for: Mechanotransductive Osteogenesis Through Microarchitectural Stabilization in an Injectable Hydrogel–Mineral System
Source: J Funct Biomater. 2026 Aug 6;17(8):389. doi: 10.3390/jfb17080389 (PMC13514390; doi:10.3390/jfb17080389)
Supplement: Supplementary file 1 [file jfb-17-00389-s001.zip › jfb-4397989-supplementary.pdf]

Supplemntary Materials

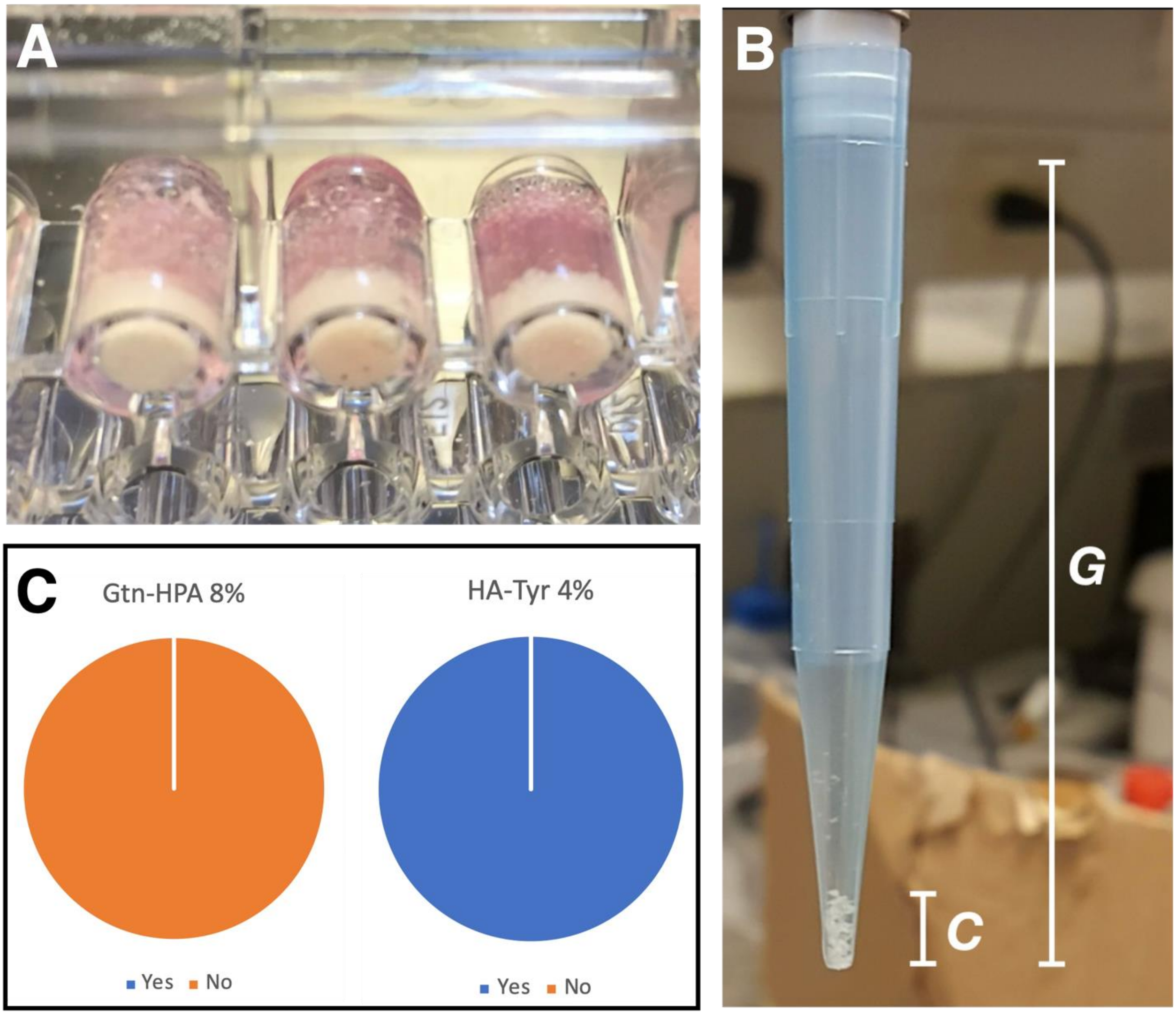

Figure S1. Sedimentation phenomenon (A) Pre-gelation CaP sinking phenomenon; (B) Quantitative analysis of clogging issue (C = vertical distance of sinking CaPs, G = total vertical distance of gel); (C) Inclusion pie graphs (n=6).

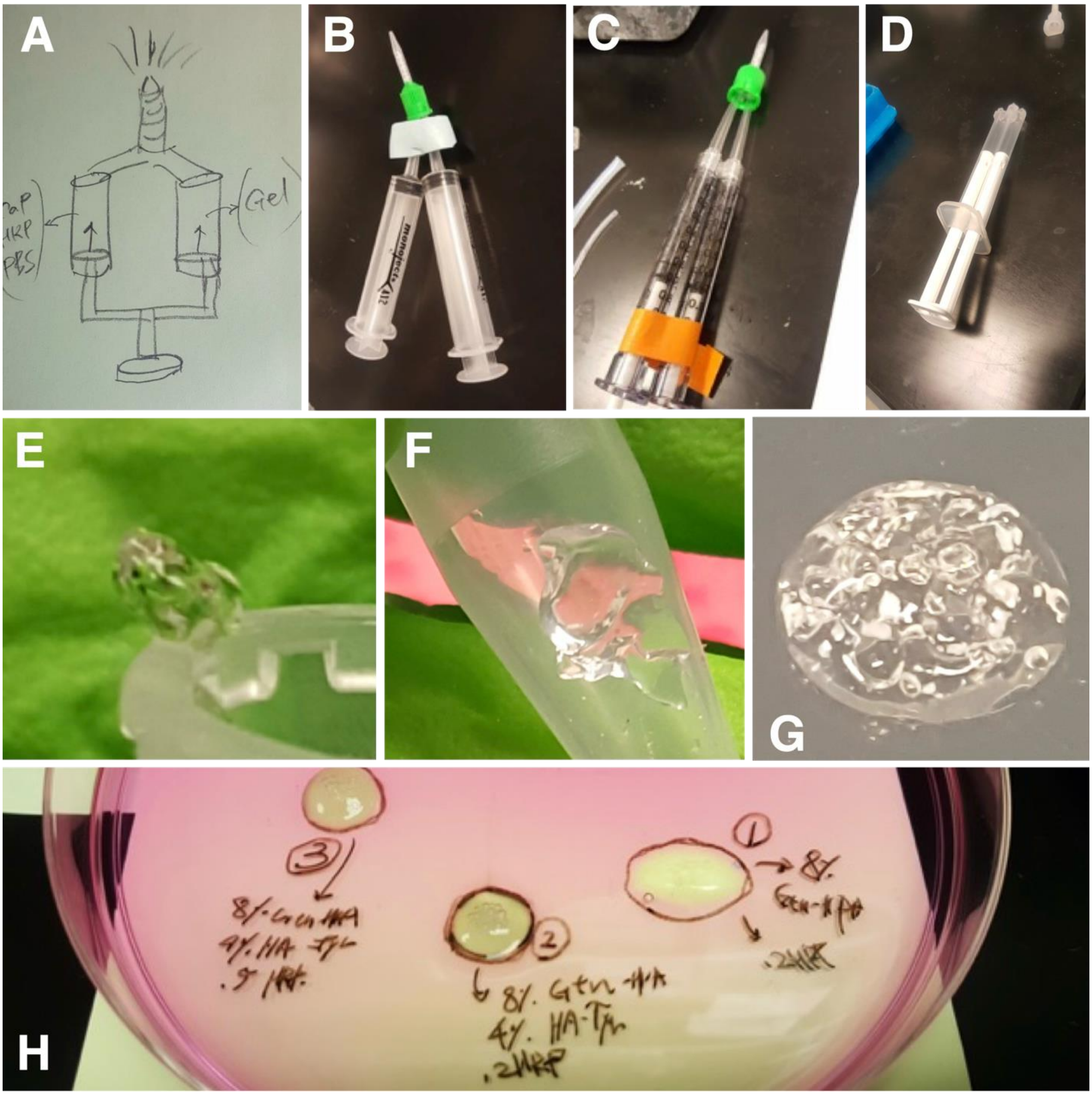

Figure S2. (A) Schematic drawing of the dual syringe model; (B) First prototype; (C) Second prototype; (D) Final syringe system; (E) Too rigidly cross-linked GH gel; (F) Too weakly cross-linked GH gel; (G) Cross-linked GH-CP; (H) Fine tuning [HRP] and [H_2O_2].

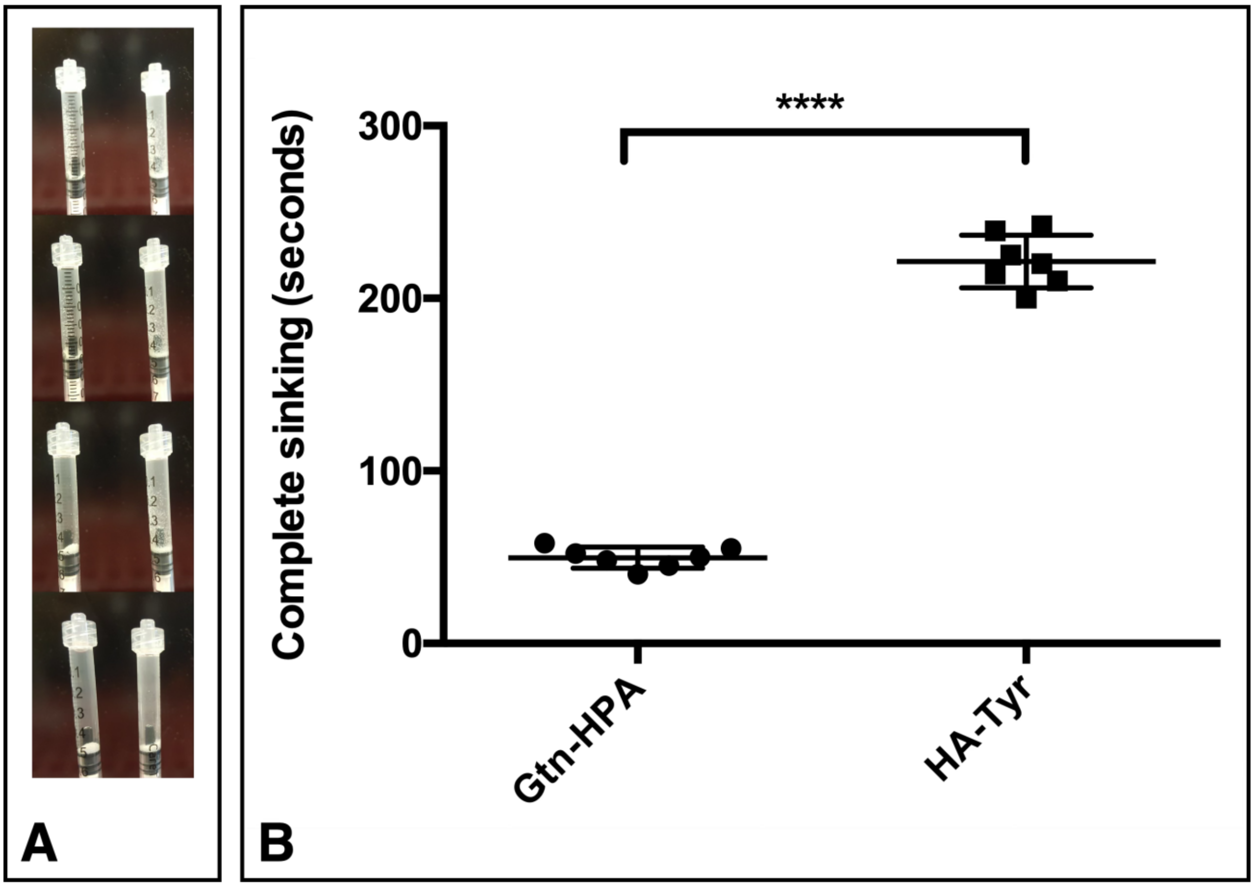

Figure S3. (A) Sequential experimental photographs of pre-gelation CaP sedimentation test; (B) Complete sinking seconds (*n*=7): Unpaired t-test, *n*=7, ****p-value < .0001.
